# Risk assessment of organ transplant operation: A fuzzy hybrid MCDM approach based on fuzzy FMEA

**DOI:** 10.1371/journal.pone.0299655

**Published:** 2024-05-23

**Authors:** Amir Sabripoor, Rouzbeh Ghousi, Mehdi Najafi, Farnaz Barzinpour, Ahmad Makuei

**Affiliations:** 1 School of Industrial Engineering, Iran University of Science and Technology, Tehran, Iran; 2 Ted Rogers School of Management, Toronto Metropolitan University, Toronto, ON, Canada; 3 Department of Industrial Engineering, Sharif University of Technology, Tehran, Iran; Istanbul University: Istanbul Universitesi, TURKEY

## Abstract

Nowadays, most fatal diseases are attributed to the malfunction of bodily. Sometimes organ transplantation is the only possible therapy, for instance for patients with end-stage liver diseases, and the preferred treatment, for instance for patients with end-stage renal diseases. However, this surgical procedure comes with inherent risks and effectively managing these risks to minimize the likelihood of complications arising from organ transplantation (maximizing life years from transplant and quality-adjusted life years) is crucial. To facilitate this process, risk ranking is used to identify and promptly address potential risks. Over recent years, considerable efforts have been made, and various approaches have been proposed to enhance Failure Modes and Effects Analysis (FMEA). In this study, taking into account the uncertainty in linguistic variables (F-FMEA), we introduce an approach based on Fuzzy Multi Criteria Decision Making (F-MCDM) for effectively evaluating scenarios and initial failure hazards. Nevertheless, the results of ranking failure modes generated by different MCDM methods may vary. This study is a retrospective study that suggests a comprehensive unified risk assessment model, integrating multiple techniques to produce a more inclusive ranking of failure modes. Exploring a broad spectrum of risks associated with organ transplant operations, we identified 20 principal hazards with the assistance of literature and experts. We developed a questionnaire to examine the impact of various critical factors on the survival of transplanted organs, such as irregularities in immunosuppressive drug consumption, inappropriate dietary habits, psychological disorders, engaging in strenuous activities post-transplant, neglecting quarantine regulations, and other design-related factors. Subsequently, we analyzed the severity of their effects on the durability of transplanted organs. Utilizing the Mamdani algorithm as a fuzzy inference engine and the Center of Gravity algorithm for tooling, we expressed the probability and severity of each risk. Finally, the failure mode ranking obtained from the F-FMEA method, three fuzzy MCDM methods, and the proposed combined method were identified. Additionally, the results obtained from various methods were evaluated by an expert team, demonstrating that the highest consistency and effectiveness among different methods are attributed to the proposed method, as it achieved a 91.67% agreement with expert opinions.

## 1. Introduction

The choice of investigating organ transplantation as the subject matter for this paper is driven by the recognition that unaddressed risks inherent in the procedure can give rise to critical situations, compelling immediate responses in hospital settings and health emergencies [1]. Over the last few years, organ transplantation has become a significant and effective method of treating patients who are at risk of losing part of their organs due to organ failure [2]. When an organ stops functioning properly, any treatment or medication can improve the function, the only way to cure is organ transplantation [3].

As a means of treating diseases associated with organ failure, organ transplants have helped thousands of patients around the world and Iran is no exception [3,4]. However, there are still factors that make it possible for a transplant operation to fail [4]. Over time, transplant recipients lose their ability to bleed because of the cold ischemia time (the maximum time an organ can survive outside the body), which decreases their chances of a successful transplant and reduces their chances of survival after transplantation thus management of ischemia time is a major challenge in the organ transplant system [5,6]. Following organ transplants, the immune systems of recipients may identify and attack the transplanted organs, viewing them as foreign entities. To mitigate the risk of rejection, post-surgery, anti-immune medications are employed to suppress the immune response. Nevertheless, there are situations where these medications may not effectively prevent rejection, resulting in a potential loss of the transplanted organ [7]. While in others, the risk of infectious diseases increases when the immune system is compromised. Because the patient that use these drugs with immunosuppressive drugs, after interaction (to prevent transplant rejection) have drug interactions and treatment of this disease in organ recipients is very difficult and, in some cases, not effective and leads to death, it is possible that it is transmitted from a transplanted organ to the recipient’s body, or that the recipient is infected with the disease from the beginning or after the transplant. In order to eliminate this risk of death after transplantation, laboratory research and timely diagnosis of this incident in the transplanted organ or recipient before transplantation are beneficial by treating patients with this preoperative disease [8]. Studies have shown that continued use of these drugs after transplantation increases the risk of cardiovascular disease and cancer, further decreasing transplant survival and death [9]. Cancer is the most prevalent complication in transplant recipients compared to other patients, which is associated with higher mortality rates and a reduced post-transplant survival rate [10,11].

The subsequent factors explain why the transplantation was declined, a study conducted revealed that high blood creatinine levels could also contribute to transplant rejection and sometimes affect the transplant organ’s quality. The results of medical studies indicate that people with high creatinine levels and who are receiving a brain-dead transplant are at greater risk of rejection and that blood creatinine levels are elevated when they have a history of blood transfusions, kidney transplants, or dialysis from brain-dead donors [11]. Reducing creatinine levels in the donor and performing transplants from living donors reduces the risk of transplant rejection. In some cases, the transplant operation failed and the transplant was rejected due to reduced organ quality [11,12]. To reduce the possibility of failure, nurses must have thorough and accurate knowledge of caring for brain death patients. The lack of organ donation and the importance of keeping organs healthy for transplantation make it necessary to train and care for patients with high accuracy and quality [13]. Therefore, it is essential to pay close attention to the process of caring for the brain-dead patient [14].

One of the factors affecting the survival rate of an organ transplant is the age of the donor and recipient [15]. As part of the medical research conducted by the Shiraz University of Medical Sciences about the age of the transplant, 1356 transplant recipients were identified in Shiraz Namazi Hospital’s transplant center. A higher cumulative probability of survival exists in the 20–40 years age group than in the 40–60 year age group and over 60 year age group. The risk of organ transplant failure and survival after transplant increases with increasing donor and recipient ages [16]. Additionally, Washington University Medical School examined the issue in 2017 in four age groups for liver donors: under 60, 60 to 69, 70 to 79, and over 80. A study found that survival rates and years after the recipient’s transplantation decreased with the donor’s age [17]. The University of Lübeck in Germany conducted research in connection with kidney transplantation that showed that age mismatches and organ size differences between donors and recipients have been important factors in shortened life spans after transplantation and sometimes even death following transplantation [18].

Nevertheless, the factors that lead to a failed organ transplant operation can be summarized in three stages, each characterized by its shortcomings, which leads to the rejection of the transplant and the failure of this operation, which includes: 1- Pre-organ transplant procedures 2- Intra-organ transplant procedures 3- post-organ transplant procedures. Although this study focuses on the third case, many studies focus on the first and second cases, while failing to address the final item will result in severe consequences for the patient. Every organization, whether a service organization, a production organization or a production service organization, has risk, which, if not managed, can damage both [19]. Since organ transplantation involves many risks, these risks lead to the failure of the organ in the patient’s body and a lower survival rate [20]. Therefore, it is better to get acquainted with the meaning of risks and their management in the following. ‬‬‬ There are many definitions of risk and risk management, but here are some of the most common ones: According to definition by [21], a risk is an uncertain event that affects one or more project goals when it occurs. According to [22], risk management is a systematic process that allows individual risk events and overall project risk to be well understood and preventive work done by minimizing opportunities for better project management. In risk management, there are four steps: risk Identification, risk Assessment, risk Treatment, and risk monitoring. In order to make informed decisions regarding risk, risk management must provide quantitative data as well as comprehensive and valuable information [23]. ‬‬‬‬‬‬‬‬‬‬‬‬‬‬‬‬‬‬‬‬‬‬‬‬‬‬‬‬‬‬‬‬‬‬‬‬‬‬‬‬‬‬‬‬‬‬‬‬‬‬‬‬‬‬‬‬‬‬‬‬‬‬‬‬‬‬‬‬‬‬‬‬‬‬‬‬‬‬‬‬‬‬‬‬‬‬‬‬

During the project risk management process, risk assessment is a vital element. As a result, higher and higher priority risks are identified for faster and more appropriate responses to prevent their consequences. This ranking determines the subsequent required analyses and scope of work. Over 65 methods have been introduced and evaluated for risk assessment and analysis in recent years that To assess risk, many methods are available, including Matrix Risk, Preliminary Risk Analysis (PHA), Failure Tree Analysis (FTA), Event Tree Analysis (ETA), Failure Modes, and Effects Analysis (FMEA) [24]. FMEAs are powerful and effective tools for minimizing potential hazards to systems [25]. This method was first applied to the analysis of failure modes and consequences in military products, particularly the aviation industry [25]. the first study examined the intent of utilizing FMEA and root cause analysis (RCA) technologies in health care and Nine infectious diseases were considered using the FMEA method [26]. When conducting a process or design FMEA, the Risk Priority Number (RPN) is a calculation that ranks risks from highest to lowest [27]. FMEA is most effective for identifying potential failure modes within system components, determining their causes, evaluating their effects on performance, and finally determining how to reduce the possibility of consequences and improve the ability to detect failure modes [28]. FMEA is utilized for the identification and management of risk and various issues. Global vessel navigation involves various and dangerous risks, thus [29] with FMEA highlights important warnings and provides solutions for managing navigation risks. In modern production systems, companies greatly benefit from proactively avoiding risks before they manifest. The utilization of FMEA in the automotive industry stands out as one of the most extensively applied techniques, resulting in the reduction of numerous hazards within the sector [30]. One more common use of this approach involves identifying and evaluating risks associated with industrial kitchen equipment in numerous commercial and non-commercial organizations. Its purpose is to decrease the occurrence of faulty products and improve overall product quality [31]. Furthermore, studies have been conducted using the FMEA method to identify and assess risks in areas related to the healthcare system, such as assessment the risks of fire incidents in medical facilities, assessing the blood transfusion procedures in public hospitals, determining the timing of antibiotic administration by healthcare personnel to patients, and managing medical waste and its associated risks [32–35]. To the best of our knowledge, there hasn’t been a study conducted using the FMEA method in the field of organ transplantation and the assessment of post-transplant risks. Although FMEA is very popular in various fields, the use of traditional FMEA models has many disadvantages and is questioned by many scientists [26,28]:

- The S, O and D risk factors are assessed using discrete ordinal scales. However, employing multiplication operations on ordinal scales is nonsensical. Consequently, the results derived are not only devoid of meaning but also misleading.
- The amalgamation of diverse risk factors may result in RPN, while the nature of the generated risks is different.
- The traditional FMEA model faces challenges in addressing ambiguity and uncertainty, and it is also incapable of handling linguistic variables.

FMEA can be changed from a traditional approach to a fuzzy method to solve these problems and limitations [36]. An alternative method to mitigate the identified weaknesses is to integrate it with MCDM method discussed in the literature [37,38]. In numerous research, researchers have commonly integrated a MCDM method alongside FMEA to effectively rank a range of alternatives [39,40]. Hence, by conducting a literature review and interviewing medical professionals, surgeons, and nurses specializing in organ transplantation and post-transplant care, this research aimed to identify existing risks that could influence both patient survival and the quality of life after transplantation. Utilizing the fuzzy FMEA approach, each identified risk was assigned a triangular fuzzy number for severity, occurrence probability, and detection probability. Furthermore, the fuzzy FMEA and the Mamdani efficiency algorithm were used to determine an appropriate index for the classification and importance of risks. Additionally, employing MCDM methods like fuzzy Additive Ratio Assessment (F-ARAS), fuzzy VIKOR (F-VIKOR), and fuzzy Weighted Aggregated Sum Product Assessment (F-WASPAS), we ranked the identified risks. Subsequently, the results obtained from various methods were compared, and a hybrid decision-making method was introduced to address inconsistencies in risk rankings due to their inherent logic. Finally, all results underwent comparison, and for evaluation, validation was conducted with transplant experts to assess the accuracy of the rankings and their outcomes. However, to the best of our knowledge, there has not been any paper published in the field of evaluating and identifying the risks post-transplant operation with A Fuzzy Hybrid MCDM Approach Based on F-FMEA.

In summary, the main innovations introduced in this study, in comparison to prior research, can be outlined as follows:

- Utilizing the FMEA methodology and MCDM method for the ranking of post-transplant risks to improve both survival and quality life years from transplant.
- Developing a novel hybrid MCDM approach based on fuzzy FMEA for the risk ranking process.
- Providing a fuzzy logic approach to tackle inherent uncertainties in input parameters.

According to the existing challenges, this study aims to address the following questions:

- How rank the existing and report post-transplant risks?
- How can Fuzzy FMEA and MCDM techniques be effective in the area of transplant surgery?
- How can we incorporate uncertainty and linguistic expressions into the input data for this issue?

As mentioned, all identified risks underwent evaluation by organ transplant specialists and were further assessed and analyzed with the assistance of additional experts.

The remainder of the paper is organized as follows. Section 2 review relevant research studies and locate recent research in the literature. Section 3, defines the problem under investigation in detail and discusses its corresponding assumptions. Section 4, the proposed approach used in this study is presented. Section 5, examines the case study, performance analysis, some managerial insights and model validation. Section 6, discussed about experimental results and performance analysis. Finally, research conclusion and future research directions are stated in sections 7.

## 2. Literature review

Optimization models related to transplantation have attracted particular attention in health care in recent years [1,2,4,7]. Here, we examine a few instances of ongoing research and reviews in this field.

Numerous investigations in the literature have focused on pre-transplant procedures, some of which we will mention. One of the first studies of organ allocation using simulation techniques to identify patient who need a transplant [41]. Alagoz et al. [14] conducted a study to assess organ acceptance rates in brain dead patients using the Markov process. To optimize societal well-being, suggest a multi-class fluid model for liver transplantation designed to predict the likelihood of rejection for a patient’s prospective organ [42]. Another study, proposed a mathematical planning model under uncertain conditions aimed at minimizing the overall cost and time required for transplant network design [43]. Piendgat and Salabun [44], to gauge the severity of Chronic Liver Disease, this study introduces the Characteristic Objects method as a prospective approach for multi-criteria decision-making. Additionally, a simulation experiment is conducted using the Model for End-Stage Liver Disease (MELD) as the foundational framework. Savaşer et al. [1], proposed objectives are to optimize the compatibility between transplanted organs and patients on the waiting list, presenting a unique perspective distinct from other research in organ transplantation. The goal is to identify the most suitable selection cluster within a hierarchical system involving various clusters. Kargar et al. [45], develops a mathematical framework for liver transportation and distribution, considering medical uncertainties and balancing tradeoffs among efficiency, fairness, cost, and time. A study has been done with distinct approach, aiming to establish a multilevel mathematical model and formulate an optimal prediction model for the quantity of organ transplants over time, with a preference for mitigating geographical interferences within the system. [46]. Rouhani et al. [2], proposed a location-allocation modeling strategy employing integer programming to guide strategic decision-making in the design of transplant networks, particularly addressing uncertainties related to supply and budget constraints. Also Goli et al. [47], proposed uncertainty mathematical model for location-allocation in an organ transplant network considered with another assumption. Salimian et al., [48], explored the identification of suitable healthcare device providers within sustainable organ transplantation networks and evaluated the sustainability of these suppliers using the fuzzy VIKOR and MARCOS methods. Al-ebbini [49], this study aims to develop a feature selection model using ant colony optimization and a k-nearest neighbor classifier to understand the relationship between specific features of lung transplant recipients and donors and the success of lung transplantations. Recently Jalilvand et al. [50], proposed a novel model for optimizing the organ transplant supply chain network, main goals are to minimize overall costs and to reduce unmet demands in uncertain conditions. The model determines the optimal number of facilities for each organ, the flows between them, and strategically allocates cold chain vehicles.

The studies and papers examined thus far have focused on pre-transplantation issues and research related to the period before organs are transplanted into a patient’s body. In the subsequent discussion, we delve into studies that specifically explore post-transplant measures and considerations.

Tong et al. [51], to investigate the experiences and future outlook of adolescent kidney transplant recipients post-kidney transplantation, an examination be conducted considering significant factors including age, gender, cultural background, and other specified criteria. This inquiry aims to assess the effectiveness of programs designed to enhance both psychological and medical outcomes in adolescents who have undergone kidney transplantation. Mangray and Vell [52], hypertension in kidney transplant recipients investigated and show hypertension impacts both patient survival and long-term transplant survival, and its optimal management requires careful analysis of causes and close monitoring of treatment. Scalia et al. [53], the objective of this study was to establish a system that quantifies the likelihood of transplant success based on distinct classes of identified variables. The study suggests employing the MCDM method (fuzzy TOPSIS) as a robust tool for evaluating information related to pancreatic islet transplantation, with the ultimate aim of making optimal decisions. Tielen et al. [54], in investigation into the response of drugs following renal transplantation and its correlation with both drug adherence and graft survival was conducted. Nonadherence emerges as a prevalent issue post-renal transplantation. The study aimed to explore connections between medication management, adherence patterns, and clinical outcomes. The findings indicate that early nonadherence following renal transplantation could potentially pose a risk factor for compromised graft survival in the later stages of life. Another research discussion investigated factors contributing to medication nonadherence in the context of kidney transplant immunosuppressive drugs within contemporary clinical practice. Nonadherence to post-transplant medication poses a significant challenge, potentially resulting in misdiagnosis, rejection, increased morbidity, diminished quality of life, and the risk of transplant failure or mortality [55]. Oweira et al. [56], Evaluated key factors such as age, gender, race, immunological compatibility, as well as the durations of cold and warm ischemia time, which impact the occurrence of graft rejection following kidney transplantation. These assessments serve as crucial considerations enabling healthcare professionals to effectively mitigate the risk of allograft rejection. Through a comprehensive understanding of these factors, doctors can implement targeted measures to minimize the likelihood of rejection, ultimately contributing to the enhanced survival of the transplanted graft. Recently, a study aimed to improve the recovery process following major surgical procedures, with a specific focus on renal transplantation. The objective was to consolidate existing evidence from the literature and formulate recommendations for an enhanced recovery after surgery protocol tailored specifically for individuals undergoing renal transplant procedures [57]. Detailed information about the reviewed studies can be found in Table 1.

**Table 1:** Categories of related publications.

Author	Year	Study Scope	Objective	Method	Uncertainty				Studied Organ	Validation	
					NO	YES				With Expert	Without Expert
						Fuzzy	Robust	Probabilistic			
Ruth et al. [46]	1985	**Pre-T**	Max NR	S	◾				Kidney		◾
Alagoz et al. [17]	2007	**Pre-T**	Max TEDR	MDP	◾				Liver		◾
Akan et al. [47]	2008	**Pre-T**	Max QALY Max NPDWO	FM	◾				Liver		◾
Tong et al. [54]	2011	**Post-T**	Max SAPW	SA	◾				Kidney		◾
Mangray et al. [55]	2011	**Post-T**	Min HAKT	SA	◾				Kidney		◾
Scalia et al.[56]	2011	**Post-T**	Max SR	F-TOPSIS		◾			Pancreatic	◾	
Zahiri et al. [48]	2014	**Pre-T**	Min TT Min TC	MILP		◾			NM		◾
Tielen et al. [57]	2014	**Post-T**	Max LYFT	SA	◾				Kidney		◾
Piendgat & Salabun [49]	2015	**Pre-T**	Max E	CO	◾					◾	
Belaiche et al. [58]	2017	**Post-T**	Max LYFT	SA	◾				Kidney		◾
Savaser et al. [1]	2019	**Pre-T**	Max PWIRF	IP	◾				Heart, Liver & Kidney		◾
Kargar et al. [8]	2020	**Pre-T**	Max TC Max TT Max SR-MU	MINLP				◾	Liver		◾
Mohammadi et al. [61]	2020	**Pre-T**	Max TNOS	MILP	◾				Liver		◾
Rouhani et al. [2]	2021	**Pre-T**	Max NR & Min TC	MILP		◾			Liver & Kidney		◾
Oweira et al. [59]	2022	**Post-T**	Min RNTIM	RP	◾				Kidney		◾
Goli et al. [9]	2022	**Pre-T**	Min TC	MILP		◾	◾		NM		◾
Salimian et al. [51]	2022	**Pre-T**	Max ISTTO	VIKOR MARCOS		◾			NM	◾	
Tielen et al. [57]	2023	**Post-T**	Min IPO	RP					Kidney		◾
Al-ebbini [52]	2023	**Pre-T**	MAX QALY	ACO-kNN	◾				Lung	◾	
Jalilvand et al. [53]	2023	**Pre-T**	Min TC Min NUD	MILP				◾	Heart, Liver & Lung	◾	
Our study	2023	**Post-T**	Max LYFT Max QALY Min PRTO	F-FMEA & F-ARAS F-VIKOR & F-WASPAS F-Hybrid MCDM		◾			Heart, Liver & Kidney	◾	

As illustrated in Table 1, the literature has a scarcity of research on post-transplant risk assessment. To the best of our knowledge, no paper has comprehensively investigated risks following transplantation while incorporating uncertainty. This absence includes the use of a robust questionnaire administered by field experts, along with the application of uncertainty theory and the Mamdani algorithm to identify, evaluate and fuzzy hybrid MCDM approach based on F-FMEA to rank risks.

Table 1 classifies the models according to twelve criteria that we have in the following:

***Study Scope***: **Pre-T:** Pre-Transplant**, Post-T:** Post-Transplant. ***Objective***: **NR:** Network Responsiveness**, TEDR:** Quality Adjusted Life Expectancy**, QALY:** Quality Adjusted Life Years**, NPDWO:** Number of Patient Deaths while Waiting for Organ**, SAPW:** Social Adjustment and Psychological Well-being**, HAKT:** Hypertension After Kidney Transplant**, TT:** Total Time**, TC:** Total Cost**, LYFT:** Life Years From Transplant**, E**: Equality**, PWIRF:** Potential Weighted Intra-Regional Flow**, SR:** Survival Rate**, MU:** Medical Urgency**, TNOS:** Total Number of Organs Shared**, RNTIM:** Risk Not Taking Immunosuppressive Medications**, ISTTO:** Improve Surgery Technology for Transplantation Operations**, IPO:** Improvements to Patient Outcomes**, PRTO:** Possibility of Rejecting Transplant Organ, **NUD**: Number of Unsatisfied Demands**. *Method*: S:** Simulation**, MDP:** Markov Decision Process**, FM:** Fluid Modeling**, SA:** Statical Analysis**, F-TOPSIS:** Fuzzy Technique for Order Preference by Similarity to Ideal Solution**, CO: Characteristic Objects, MILP:** Mixed Integer Linear Programming**, IP:** Integer Programming**, MINLP:** Mixed Integer Non-Linear Programming**, RP:** Review Paper**, VIKOR:** VlseKriterijumska Optimizacija I Kompromisno Resenje (in Serbian), **MARCOS:** Measurement Alternatives And Ranking According To The Compromise Solution**, ACO-kNN:** Ant Colony Optimization-k-Nearest Neighbor**, F-FMEA:** Fuzzy Failure Modes and Effects Analysis **F-ARAS:** Additive Ratio Assessment System**, F-WASPAS:** Weighted Aggregated Sum Product Assessment**, F-Hybrid:** Fuzzy Hybrid. ***Studied Organ***: **NM:** Not Mention.

## 3. Problem definition

As previously mentioned, when an organ loses its functionality, organ transplantation is often the only viable solution. While this medical advancement is significant, improper management can lead to severe consequences for both the patient and the healthcare system. Current research has predominantly concentrated on pre and intra-operative aspects, including decisions about transplant centers, organ allocation, transportation logistics, and more. However, there is a noticeable gap in research regarding post-transplant measures that could enhance the longevity of transplanted organs and the overall quality of life for patients. This study seeks to address this gap by examining risks that emerge after the transplant operation, potentially impacting the quality of the transplanted organ or leading to rejection by the patient’s body. Crucial assumption of this study is its focus on individuals who have reached the age recognized by law as legal maturity.

**Fig 1:**
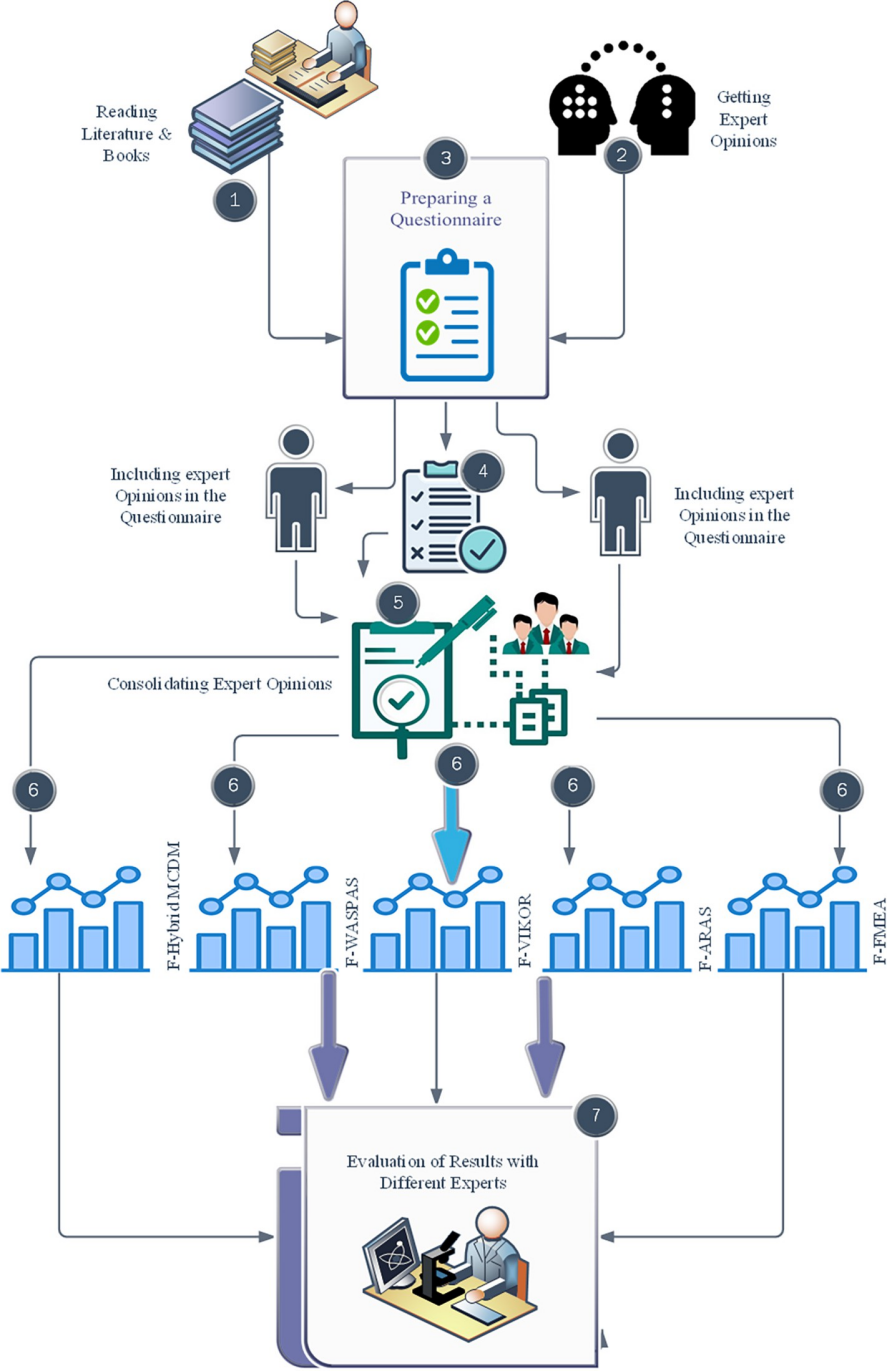
Research process flowchart.

In Fig 1 provides an overview of the comprehensive research process. This study is a retrospective study aimed at identifying influential factors in the survival of post-transplant patients. This entails the collection of information from diverse sources, including literature such as books and papers, along with consultations with nephrologists, hepatologists, and cardiologists to acquire insights into post-transplant risks. Utilizing this gathered information, the research team formulates a questionnaire designed to assess both compliance and non-compliance with various risk scenarios. This assessment takes into account factors like the probability and severity of each event, as well as the ability to detect it. Following this step, the insights provided by experts are integrated into the questionnaire. Subsequently, we utilize methodologies such as F-RPN, F-ARAS, F-VIKOR, F-WASPAS, and F-Hybrid MCDM to rank the available options and specify the ranking for each identified risk. The team subsequently consolidates these expert opinions to rank the identified risks and evaluates the outcomes. To validate the findings externally, the results are presented to three experts outside the research team, and their feedback is recorded and analyzed.

## 4. Materials and methods

This section delves into the exploration of the scope of a study that initially addresses overcoming uncertainties in the surrounding world by examining fuzzy logic. Following this, a sequential investigation unfolds, covering methodologies such as FMEA, F-FMEA, F-ARAS, F-VIKOR, F-WASPAS, and F-Hybrid MCDM approach based on F-FMEA. The content encompasses comprehensive definitions, underlying assumptions and subject matter. Ultimately, the section concludes by presenting the collective results derived from these various components.

### 4.1. Fuzzy logics

Professor Lotfali Zadeh proposed fuzzy sets in 1965 as a method to tackle the difficulties presented by uncertainty [58]. Fuzzy sets constitute an expansion of traditional set theory, wherein elements can be allocated a membership degree rather than a strict membership classification of "in" or "out" of a set [59]. This innovative approach enables the modeling of complex systems with indistinct boundaries between categories, as well as scenarios where elements can belong to multiple categories with varying degrees of membership [59]. One can summarize the rationale for employing fuzzy logic and its widespread appeal in the following manner:

I. Fuzzy logic exhibits a significant level of adaptability.
II. Fuzzy logic can accommodate data that is not suitable for traditional logic.
III. Fuzzy logic is capable of accurately modeling non-linear and complex functions.
IV. Fuzzy logic enables the direct application of expert knowledge without necessitating a training process.

There exist several types of fuzzy numbers, however, the triangular fuzzy number (TFN) is the most widely adopted among the various shapes of fuzzy numbers [60]. The following section outlines the necessary conditions for fuzzy numbers that will be utilized in the upcoming sections.

Proposition 1: If ${\tilde{\xi }}_{1}=(a,b,c)$ and ${\tilde{\xi }}_{2}=(d,e,f)$ If we have two fuzzy numbers that are in the shape of triangles, then when we add them together, the resulting fuzzy number will also be a triangular shape in Eq (1) [6,1].

$${\tilde{\xi }}_{1}⊕{\tilde{\xi }}_{2}=(a+d,b+e,c+f)$$ (1)

where *ξ*^(2)^ and *ξ*^(3)^ are the middle value and right-side value of the triangular fuzzy number $\tilde{\xi }$, respectively. Proposition 4: If *A* = (*a*_1_,*a*_2_,*a*_3_) and *B* = (*b*_1_,*b*_2_,*b*_3_) are two triangular fuzzy numbers, then their distance, denoted by d(A,B), is a triangular fuzzy number *C* = (*c*_1_,*c*_2_,*c*_3_) whose values *c*_*i*_ can be calculated using the following Eq (2) [6,1]:

$${\tilde{\xi }}_{1}⊕{\tilde{\xi }}_{2}=(a+d,b+e,c+f)$$ (2)

where in Eqs (3) and (4):

$$\left\{ \begin{array}{l} \Gamma (A)=\frac{a_{1}+4a_{2}+a_{3}}{6}\\ \Gamma (B)=\frac{b_{1}+4b_{2}+b_{3}}{6} \end{array}\right.$$ (3)

$$s_{i}=(a_{i}-\Gamma (A)+b_{i}-\Gamma (B))/2\;i=1,2,3$$ (4)

**Fig 2:**
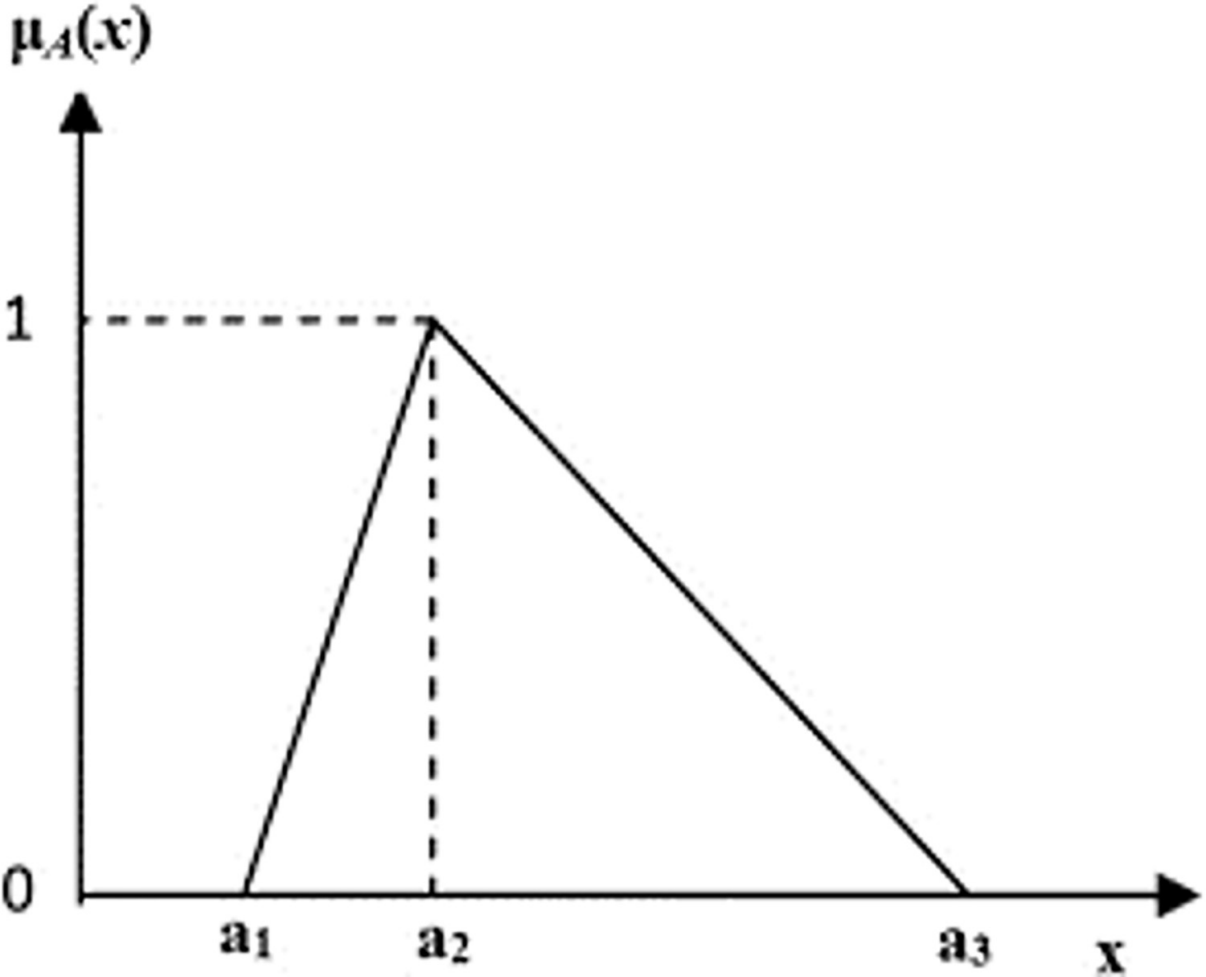
Triangle fuzzy number membership function.

Fig 2, shows an example of a fuzzy number of this kind, with a, b, and c representing the most pessimistic, the most possible, and the most optimistic respectively, furthermore, the membership function is illustrated in Eq (5).


$$\mu_{x}=\left\{ \begin{array}{ccc} 0 & \mathrm{if} & x≺a_{1}\\ \frac{x-a_{1}}{a_{2}-a_{1}} & \mathrm{if} & a_{1}\leq x\leq a_{2}\\ 1 & \mathrm{if} & x=a_{2}\\ \frac{x-a_{1}}{a_{2}-a_{1}} & \mathrm{if} & a_{2}\leq x\leq a_{3}\\ 0 & \mathrm{if} & x≻a_{3} \end{array}\right.\}$$ (5)

### 4.2. FMEA methodology

Initially, FMEA was introduced within the U.S. Armed Forces Military Strategies as FME(C)A and later found its initial application in the healthcare system during the 1990s [62], furthermore in the mid-1990s, an organization called the Foundation for Secure Medicine Honnes recommended using FMEAs to prevent errors in drug apportionment [63]. FMEA has been broadly embraced to characterize, identity, expel potential, recognized hazards and a proactive strategy that prevents system faults before they occur [62]. The first step in implementing the FMEA method to identify the risks associated with each area is to list the deficiencies, and these deficiencies with three main indicators:

**I. Occurrence (O)**:

Occurrence assesses the recurrence of a hazard or potential hazards that occur under specific conditions or in a specific environment. Occurrence is the likelihood or probability of occurrence of errors.

**II. Severity (S)**:

Severity evaluates the criticality of the consequences of a potential hazard that occurs. S-Score is evaluated based on the impact caused by the type of error.

**III. Detection (D)**:

Detection, which evaluates the probability of detecting a problem before discrimination affects the degradation to the process or framework being evaluated.

Traditional FMEA determines the RPN by multiplying these three input factors and show in Eq (6):

RPN=Occurrence(O)×Severity(S)×Detection(D) (6)

**Fig 3:**
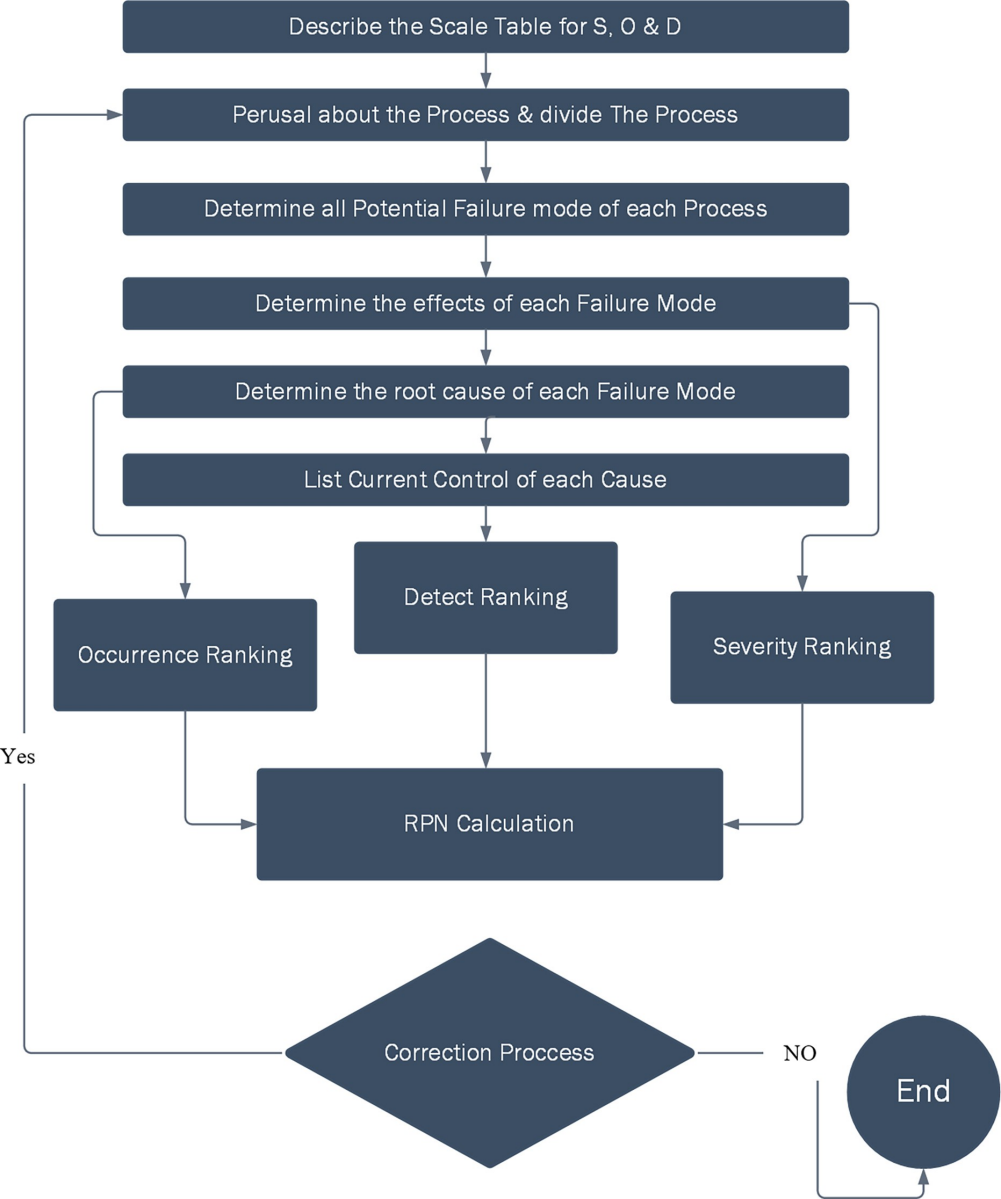
FMEA steps (Occurrence (O), Severity (S), Detection (D) and RPN).

Though FMEA was a good implementation method to avoid potential errors, the application of FMEA in the above studies revealed a limitation regarding the evaluation of RPN. The traditional FMEA operation for RPN assumes that it is divided into five levels and ratings from 1–10 to measure the probability of occurrence, severity and probability of non-detection [64]. In previous studies, assessments of RPN have indicated that scores ranging from 1 to 1000 are influenced by variations among members and industries, consequently leading to a reduction in the precision of the estimate [65]. The measures for risk assessment using traditional FMEA in the field of healthcare systems are as follows (Fig 3).

### 4.3. Fuzzy FMEA methodology

The inherent characteristics of the world give rise to numerous uncertainties, each exerting diverse impacts on different issues [66,67]. Fuzzy logic is an approach used to evaluate systems that have elements of uncertainty [68]. To improve the accuracy of prioritizing risks, the F-FMEA technique is employed instead of the traditional FMEA approach. The differentiation between these two methodologies lies in the manner of scoring each index. Traditional FMEA utilizes discrete values assigned to each item on a predefined scale, while fuzzy FMEA relies on expert input identified through a fuzzy scoring method for scoring [68]. The utilization of a fuzzy scoring system facilitated the incorporation of numerical recognition in a fuzzy manner, as elaborated further below. The expression of linguistic variables, intricate mathematical formulations, and abstract concepts in a suitable format can be challenging, especially when there is a need to categorize diverse modes of a concept [40]. Fuzzy models utilize a series of if-then rules for making inferences, and fuzzy inference engines are utilized for transforming fuzzy sets in the input space into fuzzy sets in the output space. The most common fuzzy inference engines are Mamdani and Sugeno engines [69]. Fuzzy generators provide mathematical data based on the defined membership functions for the input variables. In practical situations, an exact number is required as the system output, thus determining this undefined output is crucial. The Mamdani Max-Min Inference System is one of the many methods for fuzzy inference and is often the most convenient method [69]. The Mamdani algorithm is a widely used method for fuzzy inference in fuzzy logic systems. It involves several steps, including fuzzification, rule evaluation, aggregation, and defuzzification [70]. During the fuzzification step, crisp input values are transformed into fuzzy sets using membership functions, assigning each input variable a membership function that maps the input value to a degree of membership in the fuzzy set. The rule evaluation step involves using a set of if-then rules to determine the fuzzy output values. The antecedent of each rule is evaluated by combining the degrees of membership of the input variables using fuzzy operators such as AND, OR and NOT, while the consequent defines the fuzzy set corresponding to the output value. After evaluating all the rules, the outputs are aggregated to obtain a single fuzzy set that represents the overall output of the system, using operators such as MAX or SUM. Finally, the defuzzification step involves converting the aggregated fuzzy output set into a crisp output value by selecting a representative value from the output fuzzy set. The centroid method, which calculates the center of gravity of the output fuzzy set, is the most commonly used defuzzification method.

**Fig 4:**
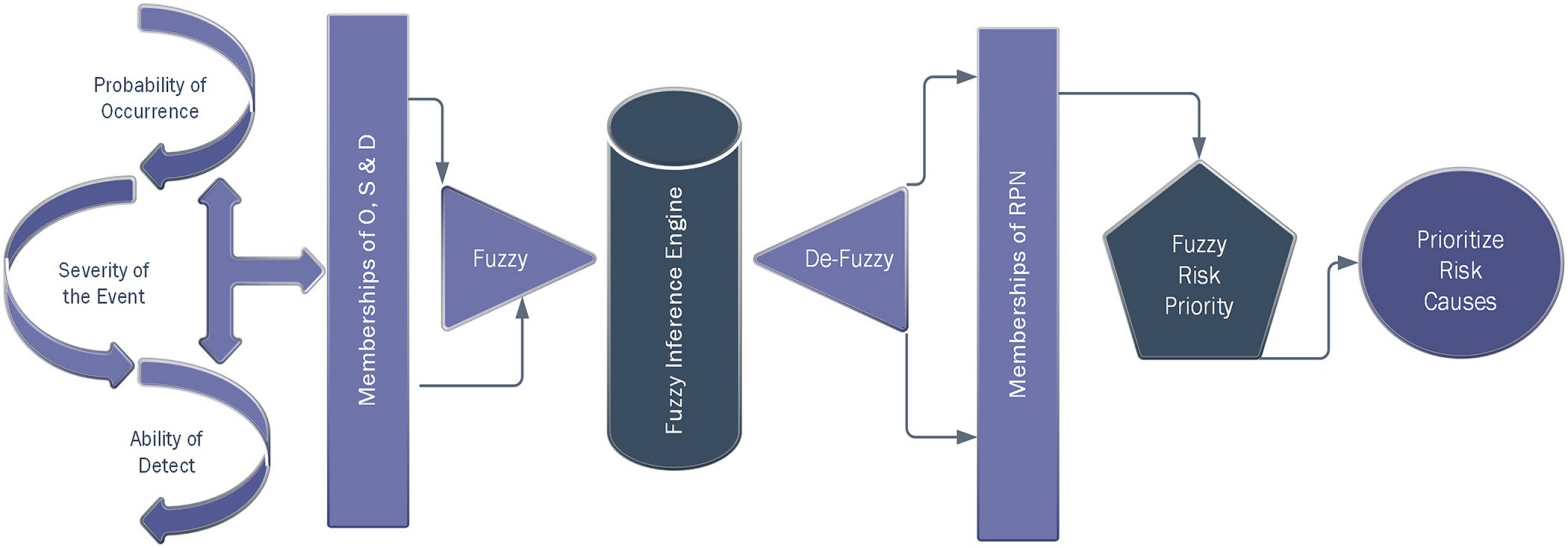
Fuzzy FMEA steps (Occurrence (O), Severity (S), Detection (D) and F-RPN).

The Mamdani algorithm is commonly acknowledged for its simplicity and ease of implementation, making it adept at managing intricate rules that encompass multiple inputs and outputs. Fig 4, displays the process of fuzzy FMEA steps.

The lexical classification criteria are presented in several Tables. Table 2, displays the likelihood of faults and imperfections using fuzzy ratings. Table 3, presents the severity of incidents using fuzzy rankings. Table 4, exhibits the fuzzy rankings of the likelihood of detection. Table 5, provides the linguistic variables used for the F-RPN in the Fuzzy FMEA method. These three factors membership function is determined through linguistic variables [70,71].

**Table 2:** Fuzzy rankings for the probability occurrence of failures and defects.

Ranking	Severity of Events	Fuzzy Number
Dangerous without warning	Extreme intensity without warning	**9,10,10))**
Dangerous with warnings	Extremely high event intensity with alert	**(8,9,10)**
very high	Destructive changes	**(7,8,9)**
high	Abnormal performance	**(6,7,8)**
Moderate	Abnormal and repairable performance	**(5,6,7)**
Low	Abnormal performance with little	**(4,5,6)**
very Low	Normal performance with reduced	**(3,4,5)**
minor	Normal performance with slight reduction	**(2,3,4)**
Very minor	Natural function with minor effects	**(1,2,3)**

**Table 3:** Fuzzy rankings for the severity of events.

Ranking	Severity of Events	Fuzzy Number
Dangerous without warning	Extreme intensity without warning	9,10,10))
Dangerous with warnings	Extremely high event intensity with alert	(8,9,10)
very high	Destructive changes	(7,8,9)
high	Abnormal performance	(6,7,8)
Moderate	Abnormal and repairable performance	(5,6,7)
Low	Abnormal performance with little	(4,5,6)
very Low	Normal performance with reduced	(3,4,5)
minor	Normal performance with slight reduction	(2,3,4)
Very minor	Natural function with minor effects	(1,2,3)

**Table 4:** Fuzzy rankings for detecting and tracking defects.

Ranking	Probability of Detecting	Fuzzy Number
Very High	Chance of discovery is very high	(1,1,3)
High	High chance of discovery	(1,3,5)
Moderate	Probability so-so	(3,5,7)
Low	Low chance by experiment	(5,7,9)
Remote	No chance to discover	(8,10,10)

**Table 5:** Linguistic variables for RPN in the fuzzy FMEA approach.

Score	VL	L	M	H	VH
**Linguistic Variable**	Very Low	Low	Moderate	High	Very High
**Fuzzy Weight**	(0,0,0.25)	(0,0.25,0.5)	(0.25,0.5,0.75)	(0.5,0.75,1)	(0.75,1,1)

However, the crucial aspect to consider is the methodology for determining the F-RPN value based on the fuzzy values of O, S, and D. The F-RPN value can be computed by multiplying the membership functions of occurrence, severity and detection according to the following equation [72]. In the case of S being a linguistic variable, its triangular fuzzy number can be described in the following manner in Eq (7):

S=(α,β,γ) (7)

**Fig 5:**
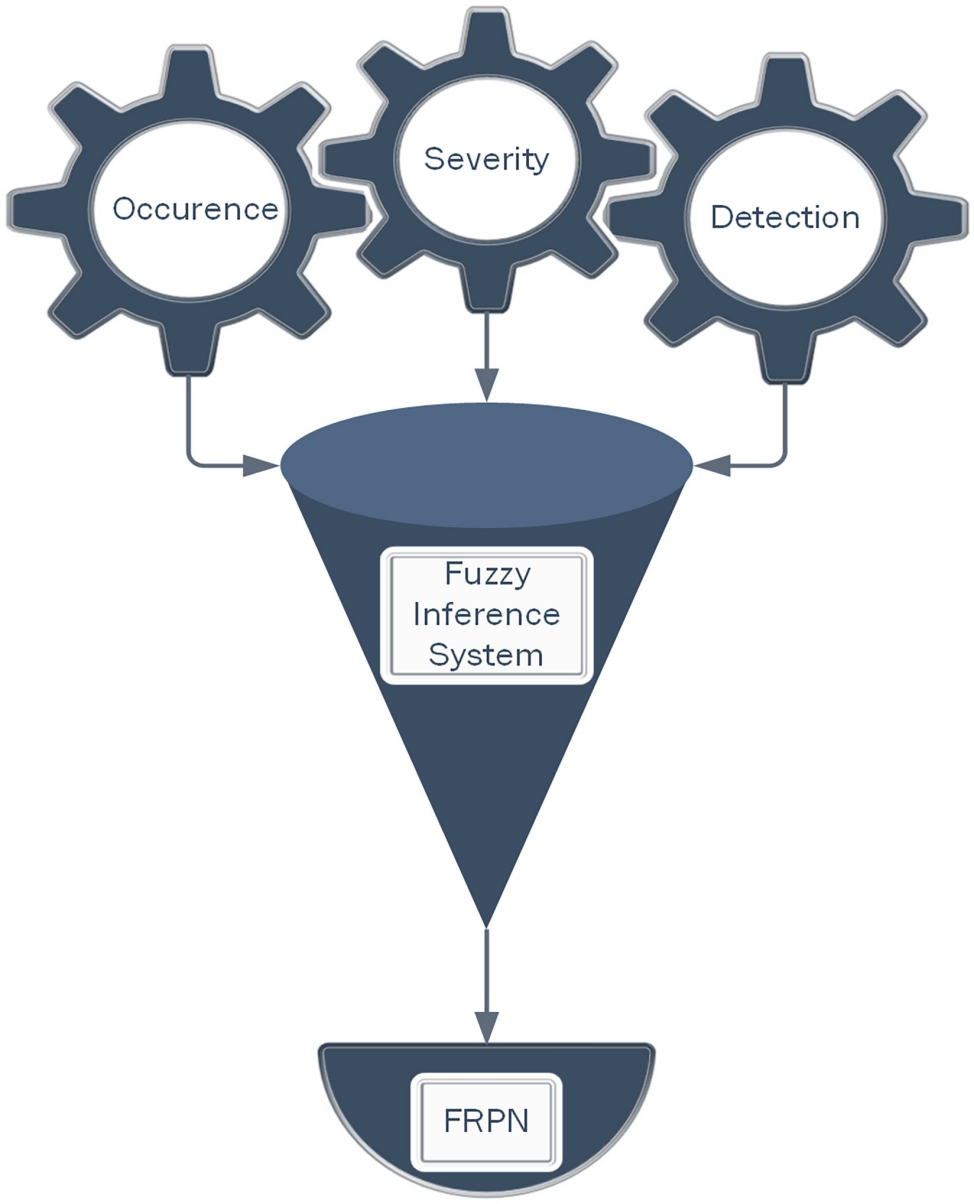
F-RPN model diagram.

The principles of algebraic operations for triangular numbers are utilized in the computation of F-RPN, calculated according to Eq (8) and the F-FRPN model diagram is described in Fig 5.


$$FRPN=S\times O\times D=(\alpha_{1},\beta_{1},\gamma_{1})\times (\alpha_{2},\beta_{2},\gamma_{2})\times (\alpha_{3},\beta_{3},\gamma_{3})=(\alpha_{1}\alpha_{2}\alpha_{3},\beta_{1}\beta_{2}\beta_{3},\gamma_{1}\gamma_{2}\gamma_{3})$$ (8)

### 4.4. Applying F-ARAS, F-VIKOR, and F-WASPAS method for the prioritization of FM

#### 4.4.1. F-ARAS

In recent years, Zavadskas and Turskis introduced a groundbreaking approach to MCDM known as the ARAS method [73]. This method, extended further as the Fuzzy ARAS, incorporates fuzzy logic to tackle uncertainties, particularly when dealing with evaluation information that spans diverse measurement units. The foundation of the ARAS method rests on the notion that decision-makers can effectively navigate the complexities of the real world through simple and direct comparisons [74]. Within this methodology, preference degrees play a crucial role in assessing the performance and desirability levels of various options relative to the existing conditions.

The ARAS method is characterized by its notable simplicity in application, streamlining the identification of a preferred function within a given context. This simplicity is accomplished by taking into account the relative values and weights assigned to the primary criteria in a specific problem. In essence, the method provides a straightforward and accessible approach to assess and prioritize criteria, making the decision-making process more efficient and user-friendly. The ARAS method, thus, offers a streamlined and efficient approach to decision-making in complex scenarios. Proceeding further, the stages of the F-ARAS method are as follow:

**Step 1:** The construction of a Fuzzy Decision-Making Matrix (FDMM) involves assigning performance values (${\tilde{x}}_{ij}$) and attribute weights (${\tilde{w}}_{j}$) as matrix entries. The selection of linguistic ratings is integral to this process. $\tilde{X}={\left[{\tilde{x}}_{ij}\right]}_{m\times n}$ capturing preferences for *m* viable alternatives (rows) evaluated across *n* attributes (columns).

**Step 2:** Determining a hypothetical ideal value. The calculated ideal values for the criteria according to Eq (9):

$${\tilde{x}}_{0j}=\{\begin{array}{c} \underset{i}{\max\;{\tilde{x}}_{ij}},if\;\underset{i}{\max\;{\tilde{x}}_{ij}}\;is\;preferable\\ \underset{i}{\min\;{\tilde{x}}_{ij}},if\;\underset{i}{\min\;{\tilde{x}}_{ij}}\;is\;preferable \end{array};i=0,1,2,\ldots ,m|j=0,1,2,\ldots ,n$$ (9)

**Step 3:** FDMM is normalized j according to Eq (10):

x¯˜ij={x˜ij∑i=0mx˜ij,ifmaxx˜ijiispreferable1x˜ij∑i=0m1x˜ij,ifminx˜ijiis.preferable;i=0,1,2,…,m|j=0,1,2,…,n (10)

**Step 4:** Calculate the weighted normalized FDMM ${\tilde{\hat{X}}}_{ij}$ according to Eq (11):

$${\left[{\tilde{\hat{X}}}_{ij}\right]}_{m\times n};{\tilde{\hat{x}}}_{ij}={\tilde{\bar{x}}}_{ij}{\tilde{w}}_{j};i=0,1,2,\ldots ,m|j=0,1,2,\ldots ,n$$ (11)

**Step 5:** Calculate values of the optimality function, total relative importance of *i*^*th*^ alternative according to Eq (12):

$${\tilde{S}}_{i}=\sum_{j=0}^{n}{\tilde{\hat{x}}}_{ij},i=0,1,2,\ldots ,m$$ (12)

It’s noteworthy that the outcomes of fuzzy performance assessment for individual alternatives are expressed as fuzzy numbers ${\tilde{S}}_{i}$. The center-of-area method emerges as the most practical and straightforward approach for the process of defuzzification and compute according to Eq (13):

$$S_{i}=\frac{1}{3}(S_{i\alpha }+S_{i\beta }+S_{i\gamma })$$ (13)

**Step 6:** The degree of utility for an alternative is ascertained through a comparison of the analyzed variant with the ideally optimal one, *S*_0_. The equation employed to compute the utility degree *K*_*i*_ for an alternative *x*_*i*_ according to Eq (14):

$$K_{i}=\frac{S_{i}}{S_{0}},i=0,1,2,\ldots ,m$$ (14)

**Step 7:** alternatives are ranked according to the value of *K*_*i*_, indicating that a higher *K*_*i*_ value corresponds to increased desirability of the option.

#### 4.4.2. F-VIKOR

The term "VIKOR" originates from Serbian and refers to a methodology designed for compromise and multi-criteria optimization [75]. The VIKOR method was initially introduced by Opricovic and subsequently expanded by Opricovic and Zeng. The F-VIKOR variant enhances its applicability by addressing uncertainties inherent in real-world situations, making it a robust tool for decision-makers dealing with incomplete or imprecise information [75].

To tackle MCDM problems characterized by conflicting and non-commensurable criteria, where compromise is deemed acceptable for conflict resolution, the decision-maker seeks a solution that closely approximates the ideal. Alternatives are evaluated based on all established criteria. In summary, the VIKOR method is notable for its versatility, transparency, and effectiveness in managing complex decision scenarios.

Proceeding further, the stages of the F-VIKOR method are outlined as follows:

**Step 1:** The construction of a Fuzzy Decision-Making Matrix (FDMM) involves assigning performance values (${\tilde{x}}_{ij}$) and attribute weights (${\tilde{w}}_{j}$) as matrix entries. The selection of linguistic ratings is integral to this process. $\tilde{X}={\left[{\tilde{x}}_{ij}\right]}_{m\times n}$ capturing preferences for *m* viable alternatives (rows) evaluated across *n* attributes (columns).

**Step 2:** Determine the fuzzy best value (FBV) and fuzzy worst value (FWV) for the all criteria according to Eq (15):

$$\begin{array}{c} {\tilde{f}}_{j}^{*}=\underset{i}{\max}\;{\tilde{x}}_{ij},.......i=1,2,\ldots ,m\\ {\tilde{f}}_{j}^{-}=\underset{i}{\min}\;{\tilde{x}}_{ij},.......i=1,2,\ldots ,m \end{array}$$ (15)

**Step 4:** Compute the values of ${\tilde{S}}_{i}$ and ${\tilde{R}}_{i}$, in order that denotes the distance rate of *i*^*th*^ alternative to the positive ideal solution, represents the distance rate of *i*^*th*^ alternative to the negative ideal solution according to Eqs (16) and (17):

$${\tilde{S}}_{i}=\sum_{j=1}^{n}\frac{{\tilde{w}}_{j}({\tilde{f}}_{j}^{*}-{\tilde{x}}_{ij})}{\left({\tilde{f}}_{j}^{*}-{\tilde{f}}_{j}^{-}\right)}$$ (16)

$${\tilde{R}}_{i}=\underset{j}{\max}[\frac{{\tilde{w}}_{j}({\tilde{f}}_{j}^{*}-{\tilde{x}}_{ij})}{\left({\tilde{f}}_{j}^{*}-{\tilde{f}}_{j}^{-}\right)}]$$ (17)

**Step 5:** Compute the values ${\tilde{Q}}_{i}$ by the relation according to Eq (18):

$${\tilde{Q}}_{i}=\upsilon [\frac{\left({\tilde{S}}_{i}-{\tilde{S}}^{*}\right)}{\left({\tilde{S}}^{-}-{\tilde{S}}^{*}\right)}]+(1-\upsilon )[\frac{\left({\tilde{R}}_{i}-{\tilde{R}}^{*}\right)}{\left({\tilde{R}}^{-}-{\tilde{R}}^{*}\right)}]$$ (18)

Where

$$\begin{array}{cc} {\tilde{S}}^{*}=\underset{i}{\min}\{S_{i}\}, & {\tilde{S}}^{-}=\underset{i}{\max}\{S_{i}\}\\ {\tilde{R}}^{*}=\underset{i}{\min}\{R_{i}\}, & {\tilde{R}}^{-}=\underset{i}{\max}\{R_{i}\} \end{array}$$ (19)

*υ* is the weight of the strategy of “the majority of criteria” (maximum group utility”), suppose that *υ* = 0.5.

It’s noteworthy that the outcomes of fuzzy performance assessment for individual alternatives are expressed as fuzzy numbers ${\tilde{Q}}_{i},{\tilde{S}}_{i}$ and ${\tilde{R}}_{i}$. The center-of-area method emerges as the most practical and straightforward approach for the process of defuzzification according to Eqs (20)–(22):

$$Q_{i}=\frac{1}{3}(Q_{i\alpha }+Q_{i\beta }+Q_{i\gamma })$$ (20)

$$S_{i}=\frac{1}{3}(S_{i\alpha }+S_{i\beta }+S_{i\gamma })$$ (21)

$$R_{i}=\frac{1}{3}(R_{i\alpha }+R_{i\beta }+R_{i\gamma })$$ (22)

**Step 6:** Rank the alternatives, sorting by the values *Q*_*i*_,*S*_*i*_ and *R*_*i*_, in decreasing order. The results are three ranking lists, we can rank the alternatives and make good decision.

**Step 7:** If both conditions are met simultaneously, the optimal compromise solution is identified as the scheme with the lowest *Q* value in the ranking. For instance,

Condition 1: The alternative *Q*(*A*^(1)^) demonstrates a satisfactory advantage if the Q value is at an acceptable level that $Q(A^{\left(2\right)})-Q(A^{\left(1\right)})\geq \frac{1}{m-1}$, where *A*^(2)^ represents the alternative in the second position on the ranking list, and m is the total number of alternatives.

Condition 2: An alternative, Q(A(1)), is deemed stable within the decision-making process if it holds the best ranking in both *S*_*i*_ and *R*_*i*_.

**Step 8:** The best alternative is chosen by selecting *Q*(*A*^(*m*)^) as the optimal compromise solution with the minimum *Q*_*i*_ value, adhering to the specified conditions mentioned above.

#### 4.4.3 F-WASPAS

The WASPAS approach, developed by Zavadskas with his team, F-WASPA represents an extension of the original WASPAS method to overcome uncertain situations [76]. Much like its predecessor, WASPAS combines two models, namely the Weighted Sum Model (WSM) and the Weighted Product Model (WPM), operating within a fuzzy environment [76].

Through the integration of multiple models, the WASPAS method empowers decision-makers to take into account diverse perspectives and viewpoints while assessing alternatives. This capability enhances the robustness and balance of the decision-making process. Proceeding further, the stages of the F-WASPAS are as follow:

**Step 1:** The construction of a Fuzzy Decision-Making Matrix (FDMM) involves assigning performance values (${\tilde{x}}_{ij}$) and attribute weights (${\tilde{w}}_{j}$) as matrix entries. The selection of linguistic ratings is integral to this process. $\tilde{X}={\left[{\tilde{x}}_{ij}\right]}_{m\times n}$ capturing preferences for *m* viable alternatives (rows) evaluated across *n* attributes (columns).

**Step 2:** FDMM is normalized according to Eq (23):

$${\tilde{\bar{x}}}_{ij}=\{\begin{array}{c} \frac{{\tilde{x}}_{ij}}{\underset{i}{\max\;{\tilde{x}}_{ij}}},if\;\underset{i}{\max\;{\tilde{x}}_{ij}}\;is\;preferable\\ \frac{\underset{i}{\min\;{\tilde{x}}_{ij}}}{{\tilde{x}}_{ij}},if\;\underset{i}{\min\;{\tilde{x}}_{ij}}\;is\;preferable \end{array},i=1,2,\ldots ,m|j=1,2,\ldots ,n$$ (23)

**Step 3a:** Calculate the weighted normalized fuzzy decision matrix ${\tilde{\hat{X}}}_{q}$ for WSM according to Eq (24):

$${\left[{\tilde{\hat{X}}}_{q}\right]}_{m\times n};{\tilde{\hat{x}}}_{ij}={\tilde{\bar{x}}}_{ij}{\tilde{w}}_{j},i=1,2,\ldots ,m|j=1,2,\ldots ,n$$ (24)

**Step 3b:** Calculate the weighted normalized fuzzy decision matrix ${\tilde{\hat{X}}}_{p}$ for WPM according to Eq (25):

$${\left[{\tilde{\hat{X}}}_{p}\right]}_{m\times n};{\tilde{\bar{\bar{x}}}}_{ij}={\tilde{\bar{x}}}_{ij}^{{\tilde{w}}_{j}},,i=1,2,\ldots ,m|j=1,2,\ldots ,n$$ (25)

**Step 4:** Calculate values of the optimality function, total relative importance of *i*^*th*^ alternative according to Eq (26):

$${\tilde{Q}}_{i}=\sum_{j=1}^{n}{\tilde{\bar{x}}}_{ij},i=1,2,\ldots ,m$$ (26)

$${\tilde{P}}_{i}=\prod_{j=1}^{n}{\tilde{\bar{\bar{x}}}}_{ij},i=1,2,\ldots ,m$$ (27)

It’s noteworthy that the outcomes of fuzzy performance assessment for individual alternatives are expressed as fuzzy numbers ${\tilde{Q}}_{i}$ and ${\tilde{P}}_{i}$. The center-of-area method emerges as the most practical and straightforward approach for the process of defuzzification according to Eqs (28) and (29):

$$Q_{i}=\frac{1}{3}(Q_{i\alpha }+Q_{i\beta }+Q_{i\gamma })$$ (28)

$$P_{i}=\frac{1}{3}(P_{i\alpha }+P_{i\beta }+P_{i\gamma })$$ (29)

**Step 5:** The calculated value of the integrated utility function for an alternative using the F-WASPAS method can be ascertained through according to Eq (30):

$$K_{i}^{\prime }=\lambda Q_{i}+(1-\lambda )P_{i},0\leq \lambda \leq 1$$ (30)

It’s noteworthy that *λ* equation’s value is derived from the provided formula according to Eq (31), nevertheless, it is important to note that in the majority of research studies, this value is typically assumed to be 0.5.


$$\lambda =\frac{\sum_{i=1}^{n}P_{i}}{\sum_{i=1}^{n}Q_{i}+\sum_{i=1}^{n}P_{i}}$$ (31)

**Step 6:** alternatives are ranked according to the value of K, indicating that a higher K value corresponds to increased desirability of the option.

### 4.5. Hybrid MCDM-based FMEA model

Considerable focus has been dedicated to MCDM methods, resulting in the emergence of various approaches in this domain. Given that each MCDM method in the literature utilizes distinct logic for ranking available options in decision problems, it is common to observe divergent outcomes when applying multiple MCDM methods to the same problem. The ranking of options is contingent on the chosen approach [77]. While it is feasible to come across similar rankings across different methods for a specific problem, such instances are infrequent due to the limited commonality in logic among diverse methods.

In this study, we introduced a novel approach by amalgamating three established MCDM methods based on utility functions and similarity and in accordance with the logical principles, the TOPSIS method was developed. This was achieved through various approaches, culminating in the creation of a novel method for ranking options. It is important to highlight that an effective integration method is vital for assessing the ultimate desirability score for each alternative. Subsequently, we proceed to elaborate on the specifics and steps of the proposed methodology.

**Step 1:** All scores for identified failure cases must fall within the range of 0 to 1. The ranking indexes of ARAS, VIKOR and WASPAS are denoted by *K*_*i*_, *Q*_*i*_ and *K*_*i*_′, respectively. The noteworthy point is that the ranking obtained the VIKOR method, given VIKOR’s ranking index lower is better, in order to is transformed into an efficiency index (1−*Q*_*i*_) for our purposes.

**Step 2:** Computing the Fuzzy Positive Ideal Solution (FPIS) and Fuzzy Negative Ideal Solution (FNIS) This method entails the utilization of maximum (*η*^+^) and minimum (*η*^−^) values according to Eqs (31) and (32):

$$\eta^{+}=\underset{C}{\max}\{ARAS_{C},VIKOR_{C},WASPAS_{C}\}$$ (31)

$$\eta^{-}=\underset{C}{\min}\{ARAS_{C},VIKOR_{C},WASPAS_{C}\}$$ (32)

**Step 3:** Computing the distance of between each failure mode with FPIS and FNIS, the distance of each failure mode from the positive ($\psi_{C}^{+}$) and negative ($\psi_{C}^{-}$) ideal solutions according to Eqs (33) and (34)

$$\psi_{C}^{+}=\sqrt{{\left(ARAS_{C}-x_{1}^{+}\right)}^{2}+{\left(VIKOR_{C}-x_{2}^{+}\right)}^{2}+{\left(WASPAS_{C}-x_{3}^{+}\right)}^{2}},.......c=1,2,\ldots ,m$$ (33)

$$\psi_{C}^{-}=\sqrt{{\left(ARAS_{C}-x_{1}^{-}\right)}^{2}+{\left(VIKOR_{C}-x_{2}^{-}\right)}^{2}+{\left(WASPAS_{C}-x_{3}^{-}\right)}^{2}},.......c=1,2,\ldots ,m$$ (34)

**Step 4:** The computation of the ultimate utility index (UUI) is intended for prioritizing available options considering MCDM. In this methodology, for ranking the options, we take into account the separation distance from both the positive ideal solution and negative ideal solution using three MCDM methods, according to Eq (35):

UUI=(ψC−∑C=1mψC−)−(ψC+∑C=1mψC+),.........−1≤UUI≤1 (35)

**Step 6:** Arrange the alternatives in a descending order according to UUI, signifying that a higher UUI value corresponds to a more favorable option.

### 4.6. Participants

This study aims to recognize and assess the risk factors impacting the survival of patients post-transplantation, subsequently diminishing both patient longevity and the quality of life in the years following the transplant. In the participant selection process for this research, we employ the following five criteria:

(A). Examination of academic credentials and educational background, encompassing the attended university, lectures, publications such as books and articles, and other disseminated materials.
(B). Evaluation of participant’s knowledge and ample experience within their respective professions and specialties.
(C). Consideration of patient satisfaction regarding the treatment, interactions with healthcare providers, and the methods provided for maintaining improved health.
(D). Choosing participants possessing specialized credentials, fellowship, and sub-specialty qualifications. Furthermore, these criteria are also utilized for selecting experts to validate the results obtained in this study.

## 5. Implementation and evaluation

In this section, we utilize a combined approach based on F-FMEA the identified risks in this domain to assess their ranking in terms of credibility and applicability. The research is conducted considering uncertainty in linguistic variables. Additionally, to validate and evaluate the results, expert scrutiny from the domain is applied. In following, we proceed to examine the case study, managerial insights and the evaluation of the paper.

### 5.1. Case study

This Study aimed to pinpoint key and essential risks associated with organ transplantation at Imam Reza Hospital. The hospital engages in collaboration with Montaserieh Hospital within the same city to enhance the efficiency of organ transplant procedures. Regular meetings are conducted to elevate the standard of surgeries, ensure secure patient transfers, and mitigate risks, all contributing to the preservation of the quality of transplanted organs.

The study opted for the fuzzy FMEA method as opposed to the traditional fuzzy scoring approach typically determined by domain experts. A team comprising five specialists in organ transplantation was assembled to compile a list of potential risks sourced from various outlets, including research, papers, and interviews with members of the transplant team, nurses, and supervisors. Importantly, verbal consent was obtained from the interviewees, who verbally affirmed their agreement. Each expert was furnished with the risk list (refer to Appendix 1 & Table 12 in S1 File), and their viewpoints were evaluated utilizing linguistic criteria and numerical recognition in the fuzzy context for three probability factors and severity. The multiplication of these factors yielded a RPN for each identified risk. The prioritization of corrective and preventive measures was based on the RPN values, with higher numbers indicating a greater urgency for action.

Subsequently, employing the F-ARAS, F-VIKOR, and F-WASPAS methods, along with the F-Hybrid approach, we evaluated and ranked the existing risks. These risks were categorized based on their severity concerning organ transplant rejection and the resulting impact. Additionally, we conducted a comparative analysis among the available methods.

### 5.2 Performance analysis

In this section, we scrutinize the outcomes of our investigation by leveraging the expertise of five professionals in transplant procedures and patient care. Distinct weights were assigned to each expert based on their backgrounds, experiences, and involvement in the field. Data collection involved the use of a questionnaire encompassing 20 crucial risks in the field, evaluated across three primary factors: probability of occurrence, severity of effect, and detectability. Fuzzy Tables and the Mamdani algorithm served as the fuzzy inference engine to generate PRN as the output.

**Fig 6:**
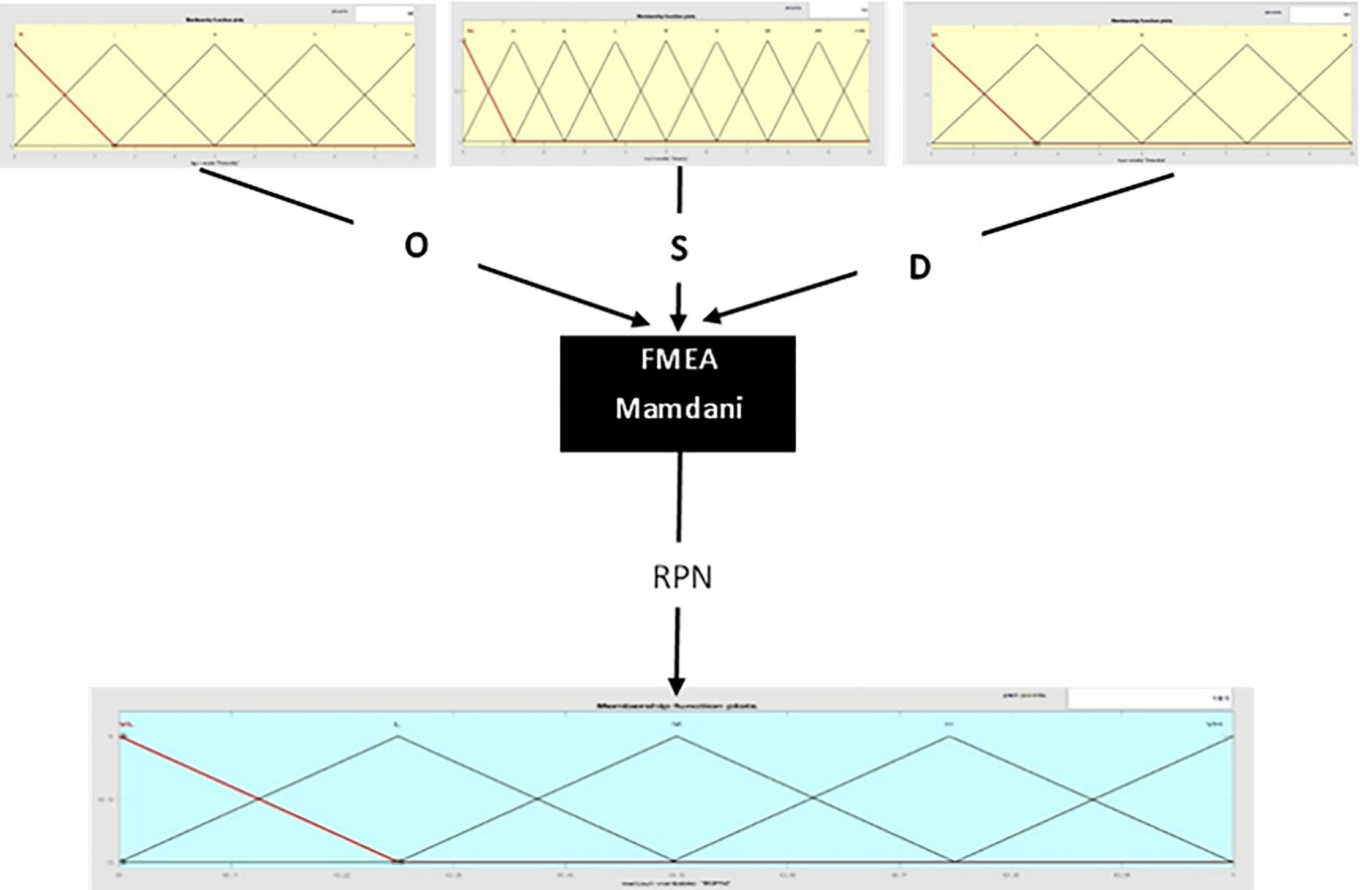
Define and combine memberships of three inputs and the RPN variable as output.

The center of gravity algorithm, along with the fuzzy toolbox in MATLAB 2020a software, was utilized for obtaining the fuzzy output in Table 6, depicted schematically in Fig 6.

**Table 6:** Failure modes identified and F-FMEA output.

	Failure modes	Cause of failure	Disabled risk	O	S	D	F- FMEA output
FM1	Irregular consumption of immunosuppressive drugs	Failure on the part of the recipient to follow medical advice	Rejection of transplant / reduction of quality of transplanted organ	Very High	Dangerous without warning	Low	VH 0.930
FM2	Possibility of post-transplant diseases	Taking anti-transplant rejection drugs	Rejection of the transplant / reduction of the quality of the transplanted organ / death of the recipient	Moderate	Very High	Moderate	VH 0.911
FM3	Ignoring post-transplant quarantine rules	Failure on the part of the recipient to follow medical advice	Rejection of transplant / reduction of quality of transplanted organ	Moderate	Very High	Moderate	VH 0.911
FM4	Error in medical research before transplantation	Negligence of the laboratory and the transplant team member.	Rejection of the transplant / reduction of the quality of the transplanted organ / death of the recipient	Low	Dangerous with warnings	Moderate	H 0.750
FM5	Donor creatine levels	Decreased blood flow, dehydration, diet, negligence of transplant and laboratory team members	Rejection of the transplant / reduction of the quality of the transplanted organ / death of the recipient	High	High	M	H 0.750
FM6	The ischemic time of the transplanted organ has elapsed	Mismanagement planning and type of organ allocation in network	Rejection of the transplant / reduction of the quality of the transplanted organ / death of the recipient	High	Very High	Low	VH 0.750
FM7	Do heavy work after transplantation	Failure on the part of the recipient to follow medical advice	Rejection of transplant / reduction of quality of transplanted organ	Remote	High	Moderate	H 0.750
FM8	Mental illness	Patients’ conscience agony	Rejection of transplant / reduction of quality of transplanted organ	High	High	Very Low	H 0.750
FM9	Improper diet	Failure on the part of the recipient to follow medical advice	Rejection of transplant / reduction of quality of transplanted organ	High	High	Very Low	H 0.750
FM10	Possibility of oral diseases	Taking anti-transplant rejection drugs	-	Moderate	High	Low	L 0.500

	**Failure modes**	**Cause of failure**	**Disabled risk**	**O**	**S**	**D**	**F-FMEA output**
FM11	Risk of Cancer, Particularly Skin Cancer and Lymphoma	Taking anti-transplant rejection drugs	Decreased quality of transplanted organ	High	High	Low	VH 0.930
FM12	The possibility of contracting cytomegalovirus	Taking anti-transplant rejection drugs	Rejection of transplant / reduction of quality of transplanted organ	Moderate	Very High	Moderate	VH 0.911
FM13	Probability of contracting viral infections	Taking anti-transplant rejection drugs	Rejection of the transplant / reduction of the quality of the transplanted organ / death of the recipient	Moderate	Very High	Moderate	VH 0.911
FM14	Weight Gain	Taking anti-transplant rejection drugs	Rejection of transplant / reduction of quality of transplanted organ	Moderate	High	Moderate	H 0.750
FM 15	Nursing mistakes	Failure on the part of the recipient to follow medical advice	Rejection of transplant / reduction of quality of transplanted organ	Low	Very High	High	H 0.750
FM16	Becoming pregnant shortly following the transplant	Failure on the part of the recipient to follow medical advice	Rejection of transplant / reduction of quality of transplanted organ	Low	High	Low	VH 0.750
FM17	Decreased quality of transplanted organ outside the body	Lack of technology to simulate the body environment outside the body	Rejection of transplant / reduction of quality of transplanted organ	Low	High	Moderate	H 0.750
FM18	Recipient and donor age	Weakening of cellular tissues	Rejection of transplant / reduction of quality of transplanted organ	Low	High	Very Low	H 0.750
FM19	Impaired physical function of the body after transplantation	Taking anti-transplant rejection drugs	Feeling weak in body function / decreased desire	Moderate	Low	Very Low	H 0.750
FM20	Inadequate physical activity and limited mobility	Failure on the part of the recipient to follow medical advice	Rejection of transplant / reduction of quality of transplanted organ	Low	Moderate	Low	L 0.500

	**Failure modes**	**Cause of failure**	**Disabled risk**	**O**	**S**	**D**	**F- FMEA output**
FM1	Irregular consumption of immunosuppressive drugs	Failure on the part of the recipient to follow medical advice	Rejection of transplant / reduction of quality of transplanted organ	Very High	Dangerous without warning	Low	VH 0.930
FM2	Possibility of post-transplant diseases	Taking anti-transplant rejection drugs	Rejection of the transplant / reduction of the quality of the transplanted organ / death of the recipient	Moderate	Very High	Moderate	VH 0.911
FM3	Ignoring post-transplant quarantine rules	Failure on the part of the recipient to follow medical advice	Rejection of transplant / reduction of quality of transplanted organ	Moderate	Very High	Moderate	VH 0.911
FM4	Error in medical research before transplantation	Negligence of the laboratory and the transplant team member.	Rejection of the transplant / reduction of the quality of the transplanted organ / death of the recipient	Low	Dangerous with warnings	Moderate	H 0.750
FM5	Donor creatine levels	Decreased blood flow, dehydration, diet, negligence of transplant and laboratory team members	Rejection of the transplant / reduction of the quality of the transplanted organ / death of the recipient	High	High	M	H 0.750
FM6	The ischemic time of the transplanted organ has elapsed	Mismanagement planning and type of organ allocation in network	Rejection of the transplant / reduction of the quality of the transplanted organ / death of the recipient	High	Very High	Low	VH 0.750
FM7	Do heavy work after transplantation	Failure on the part of the recipient to follow medical advice	Rejection of transplant / reduction of quality of transplanted organ	Remote	High	Moderate	H 0.750
FM8	Mental illness	Patients’ conscience agony	Rejection of transplant / reduction of quality of transplanted organ	High	High	Very Low	H 0.750
FM9	Improper diet	Failure on the part of the recipient to follow medical advice	Rejection of transplant / reduction of quality of transplanted organ	High	High	Very Low	H 0.750
FM10	Possibility of oral diseases	Taking anti-transplant rejection drugs	-	Moderate	High	Low	L 0.500

	**Failure modes**	**Cause of failure**	**Disabled risk**	**O**	**S**	**D**	**F-FMEA output**
FM11	Risk of Cancer, Particularly Skin Cancer and Lymphoma	Taking anti-transplant rejection drugs	Decreased quality of transplanted organ	High	High	Low	**VH 0.930**
FM12	The possibility of contracting cytomegalovirus	Taking anti-transplant rejection drugs	Rejection of transplant / reduction of quality of transplanted organ	Moderate	Very High	Moderate	**VH 0.911**
FM13	Probability of contracting viral infections	Taking anti-transplant rejection drugs	Rejection of the transplant / reduction of the quality of the transplanted organ / death of the recipient	Moderate	Very High	Moderate	**VH 0.911**
FM14	Weight Gain	Taking anti-transplant rejection drugs	Rejection of transplant / reduction of quality of transplanted organ	Moderate	High	Moderate	**H 0.750**
FM 15	Nursing mistakes	Failure on the part of the recipient to follow medical advice	Rejection of transplant / reduction of quality of transplanted organ	Low	Very High	High	**H 0.750**
FM16	Becoming pregnant shortly following the transplant	Failure on the part of the recipient to follow medical advice	Rejection of transplant / reduction of quality of transplanted organ	Low	High	Low	**VH 0.750**
FM17	Decreased quality of transplanted organ outside the body	Lack of technology to simulate the body environment outside the body	Rejection of transplant / reduction of quality of transplanted organ	Low	High	Moderate	**H 0.750**
FM18	Recipient and donor age	Weakening of cellular tissues	Rejection of transplant / reduction of quality of transplanted organ	Low	High	Very Low	**H 0.750**
FM19	Impaired physical function of the body after transplantation	Taking anti-transplant rejection drugs	Feeling weak in body function / decreased desire	Moderate	Low	Very Low	**H 0.750**
FM20	Inadequate physical activity and limited mobility	Failure on the part of the recipient to follow medical advice	Rejection of transplant / reduction of quality of transplanted organ	Low	Moderate	Low	**L 0.500**

It’s noteworthy that the average of each comment was recorded as the parameter value due to discrepancies in the opinions and critiques of the experts. The incorporation of insights from the interviewed experts aimed to present scientifically documented results. Input combination involved the "AND" operator with equal importance for each of the three factors to derive F-RPN.

The data provided in Table 6 outlines a roster of 20 risks, each linked to potential issues such as organ transplant rejection, a decline in quality, or even the prospect of patient or organ recipient mortality. Subsequently, we provide a brief explanation of the failure modes mentioned in Table 6. FM1 addresses the effect issue of irregularities in the usage of immunosuppressive drugs and the dosing of prophylactic medications employed to prevent transplant rejection. FM2 examines the consequences of acquiring illnesses following transplantation, attributed to the compromised immune system, and their influence on patient survival. FM3 explores the failure to adhere to quarantine rules, underscoring the importance of compliance to protect the patient’s recovery and prevent the spread of infections to both caregivers and the broader community. FM4 underscores that shortcomings and errors in medical research can directly influence the post-transplant survival of patients. For instance, pre-transplant research can identify the most effective methods and procedures for each patient. If research is not conducted properly, it may lead to unknown side effects affecting patients, and other factors could influence their survival. FM5 delves into the impact of elevated blood creatinine levels, serving as an important indicator of potential kidney function issues post-transplant. Whether indicative of kidney disorders, inflammation, tissue damage, or urinary tract obstruction, these fluctuations are crucial considerations. Conversely, a decline in creatinine levels signals potential issues with overall kidney function or muscle health. Understanding and managing these factors are pivotal for ensuring the post-transplant survival of patients. FM6 explores how the prolonged ischemia time of an organ can result in a decline in organ quality, leading to potential side effects in patients, with significant implications for patient survival. FM7 engaging in strenuous activities post-transplant may have a direct impact on the survival of patients. Following a transplant operation, prioritizing rest and allowing the body time for recovery, along with careful care of the surgical site, is of paramount importance. FM8 Stress, depression, anxiety, and guilt can trigger the release of stress hormones, adversely affecting both physical and overall bodily functions. Patients dealing with mental health disorders may encounter difficulties in decision-making and awareness, impacting their ability to make informed choices regarding treatment options, follow post-transplant guidelines, and actively participate in the recovery process. Additionally, individuals with mental health conditions may undergo alterations in health-related behaviors, potentially leading to delays in recovery, the emergence of new challenges, and ultimately influencing the overall well-being and survival of patients significantly. FM9 Weight control serves as a protective measure for transplant patients against issues arising from heart diseases, diabetes, and high blood pressure. Adhering to a low-calorie diet helps prevent excess weight gain, as increased weight places additional strain on organs. Given the susceptibility to various infections due to the use of immunosuppressive drugs, it is crucial to maintain cleanliness in the environment and prevent the transmission of contaminants to transplant recipients. recipient organ who have undergone transplantation are significantly more prone to various diseases. Moreover, the daily consumption of specific types of food and the manner in which they are prepared play a crucial role in the overall well-being and survival of transplant patients. FM10 issues may cause medical complications after organ transplantation, as dental infections can heighten immune system activity, potentially impacting the acceptance of the transplant organ. According to FM11exposure to harmful sunlight or radiation heightens the risk of complications after transplantation. While skin cancer is not directly linked to rejection, its treatments, like surgery and chemotherapy, can impact the immune system, affecting the acceptance and sustainability of the transplanted organ. FM12 to explain how CMV infection in individuals who have undergone organ transplantation can result in severe complications. These complications encompass inflammation, issues with the kidneys, and significantly, an increased likelihood of rejecting the transplanted organ. FM13 addressed probability of contracting viral infections and effect on survival. For instance, contracting herpes simplex viruses type 1 and 2 is a possibility following a transplant procedure, potentially causing skin problems or internal infections. Cold viruses, particularly rhinoviruses, are identified as contributors to respiratory infections after organ transplantation and other viruses influence the post-transplant survival of patients. FM14 excessive weight gain, reaching a point where it poses substantial challenges to the body, contributes to serious health risks. These include heightened infection susceptibility, increased blood pressure, and diabetes, as well as disruptions in hormonal balance and adverse effects on cardiovascular function. These factors collectively impact patient survival, overall body health, and the longevity of the transplanted organ. weight gain, reaching a point where it poses substantial challenges to the body, contributes to serious health risks. These include heightened infection susceptibility, increased blood pressure, and diabetes, as well as disruptions in hormonal balance and adverse effects on cardiovascular function. These factors collectively impact patient survival, overall body health, and the longevity of the transplanted organ. FM15 medical errors can have a significant impact on the survival of transplant recipients. Mistakes in the surgical process or postoperative stages may lead to an increased risk of infections, affecting patient survival. Errors in prescribing and managing medications can result in serious side effects, influencing the outcomes of transplant procedures. The secondary traumatic consequences arising from mistakes in the surgical process can transform into side effects impacting the survival of organ transplant recipients, and directly aim to the survival of both the transplanted organ and the patients. FM16 highlights another pivotal factor influencing the survival of transplanted organs. Women who conceive after organ transplantation face significant risks. Moreover, there is a risk of organ rejection while pregnancy is feasible for transplant recipients, it is associated with elevated rates of maternal and fetal mortality, premature delivery, intrauterine growth restrictions, and congenital anomalies. FM17 investigates how the duration of storage over time affects the risks associated with cellular damage and tissue changes, potentially resulting in a deterioration of organ quality. FM18 acknowledges that age is a crucial factor affecting the survival rate in transplantation. Studies suggest that individuals between the ages of 20 and 40 are deemed optimal donors, given their potential for better cumulative graft survival. F19 explains that compromised physical function following organ transplantation can have a notable impact on both patient survival and quality of life. Such disruptions require adjustments to the individuals’ lifestyle, and the neglect of even minor issues can result in unfavorable consequences. Finally last factor (FM20) explains how insufficient physical activity and restricted mobility can exert direct and indirect impacts on the performance of a transplanted organ. Inadequate activity levels may lead to complications, including muscular and tissue damage, heightened risks of cardiovascular issues, and an increased susceptibility to thrombosis formation Furthermore, the causes of these errors, along with three key components: severity of the effect, probability of occurrence, and probability of detection has been fully presented. The product of these factors leads to the determination of the RPN for every failure mode, each being itemized and calculated separately.

Following this, we computed the K index in the F-ARAS method, the Q index in the F-VIKOR method, the K index in the F-WASPAS method and the rankings of the failure modes generated using three MCDM methods detailed in Table 7.

**Table 7:** Results of the three MCDM methods.

Method	ARAS		VIKOR		WASPAS	
	*K*_*i*_	Rank	1−*Q*_*i*_	Rank	*K*_*i*_′	Rank
FM 1	1	1	0.886001	1	0.908328	1
FM 2	0.639173	9	0.490006	5	0.61668	6
FM 3	0.639173	8	0.490006	6	0.61668	6
FM 4	0.547233	16	0.227734	12	0.525401	14
FM 5	0.641408	7	0.256854	10	0.574432	12
FM 6	0.820733	2	0.788539	2	0.768887	2
FM 7	0.449923	19	0.023046	20	0.427602	19
FM 8	0.597983	13	0.094016	17	0.505932	15
FM 9	0.597983	13	0.097349	16	0.505932	15
FM 10	0.697882	5	0.52666	4	0.669031	4
FM 11	0.800098	3	0.653339	3	0.746237	3
FM 12	0.639173	9	0.490006	6	0.61668	6
FM 13	0.639173	9	0.490006	6	0.61668	6
FM 14	0.618537	12	0.443694	9	0.596717	10
FM 15	0.41857	20	0.060223	19	0.408149	20
FM 16	0.556623	15	0.170121	13	0.527339	13
FM 17	0.477278	18	0.097572	15	0.464974	18
FM 18	0.656759	6	0.250875	11	0.594369	11
FM 19	0.732604	4	0.158472	14	0.661489	5
FM 20	0.519892	17	0.085798	18	0.488624	17

In Table 8, the metrics for each method have been computed, and using these metrics, the available options (failure mode) have been arranged in order of priority. Continuing with the information from Table 7, we have Table 8, showcasing the outcomes of the F-Hybrid MCDM method.

**Table 8:** Results of the proposed F-Hybrid MCDM method.

	$\psi_{C}^{+}$	$\psi_{C}^{-}$	*UUI*	Rank
FM 1	0	1.154527	0.132745	1
FM 2	0.609973	0.556959	0.022556	7
FM 3	0.609973	0.556959	0.022556	7
FM 4	0.885972	0.2687	-0.02936	13
FM 5	0.797434	0.363282	-0.01246	12
FM 6	0.247143	0.936935	0.09092	2
FM 7	1.130652	0.036898	-0.07265	20
FM 8	0.97508	0.216304	-0.04144	16
FM 9	0.972375	0.217421	-0.04113	15
FM 10	0.526939	0.63222	0.036856	4
FM 11	0.346939	0.81064	0.069612	3
FM 12	0.609973	0.556959	0.022559	5
FM 13	0.609973	0.556959	0.022556	6
FM 14	0.662005	0.502484	0.012754	9
FM 15	1.12701	0.037177	-0.07237	19
FM 16	0.92424	0.2343	-0.03592	14
FM 17	1.044711	0.110589	-0.05833	18
FM 18	0.787255	0.378574	-0.01001	11
FM 19	0.813468	0.425604	-0.00639	10
FM 20	1.02322	0.143807	-0.05305	17
∑	14.70433	8.697298	0.132745	------------

Examining Tables 7 and 8, these Tables depict the prioritization of current risks based on occurrence, impact severity, and probability criteria. Table 7 employs a unified method for ranking risks, while Table 8 focuses on implementing the proposed method and combining introduced approaches to ensure robust outcomes. To elaborate further, Table 7 documents the final output and index for evaluating the significance of the identified 20 risks in each column, along with the rank of each risk within that method among the existing risks. In Table 8, considering its procedural steps in Eqs 31–35, it computes the FPIS and FNIS indices. Ultimately, through the calculation of the UUI index, it assigns rankings to the existing risks and suggested as an approach to integrate and combine decision-making methods for a more resilient and trustworthy ranking.

The examinations, classifications, reviews of identification and ranking methods, as well as analysis performed have been carried out within the managerial insights section.

### 5.3. Managerial insights

The proposed approach for the identification, control, and analysis of existing risks in various domains such as healthcare, services, and industrial sectors is well-suited for practical application. A literature review in this field, coupled with insights from experts and experts in organ transplant field, plays a pivotal role in identifying prevalent post-transplant risks. Furthermore, incorporating considerations for uncertainties and linguistic variables is imperative to fortify the robustness of the results, all of these ensuring not only patient survival but also an enduring enhancement in the quality of life over an extended period. Based on the criteria of impact severity, occurrence, and probability of failure, the ultimate ranking of the 20 identified risks is determined using F-FMEA with F-RPN score, F-ARAS, F-VIKOR, F-WASPAS and the proposed method, as outlined in according to Tables 6–8, the leading five fault modes are outlined in Table 9.

**Table 9:** The ranking leading five fault methods by the five methods.

Methods	Leading Five Fault Modes
F-FMEA	FM1≻FM6≻FM11≻FM10≻FM19
F-ARAS	FM1≻FM6≻FM11≻FM19≻FM10
F-VIKOR	FM1≻FM6≻FM11≻FM10≻FM2
F-WASPAS	FM1≻FM6≻FM11≻FM10≻FM19
Proposed Method	FM1≻FM6≻FM11≻FM10≻FM12

**Fig 7:**
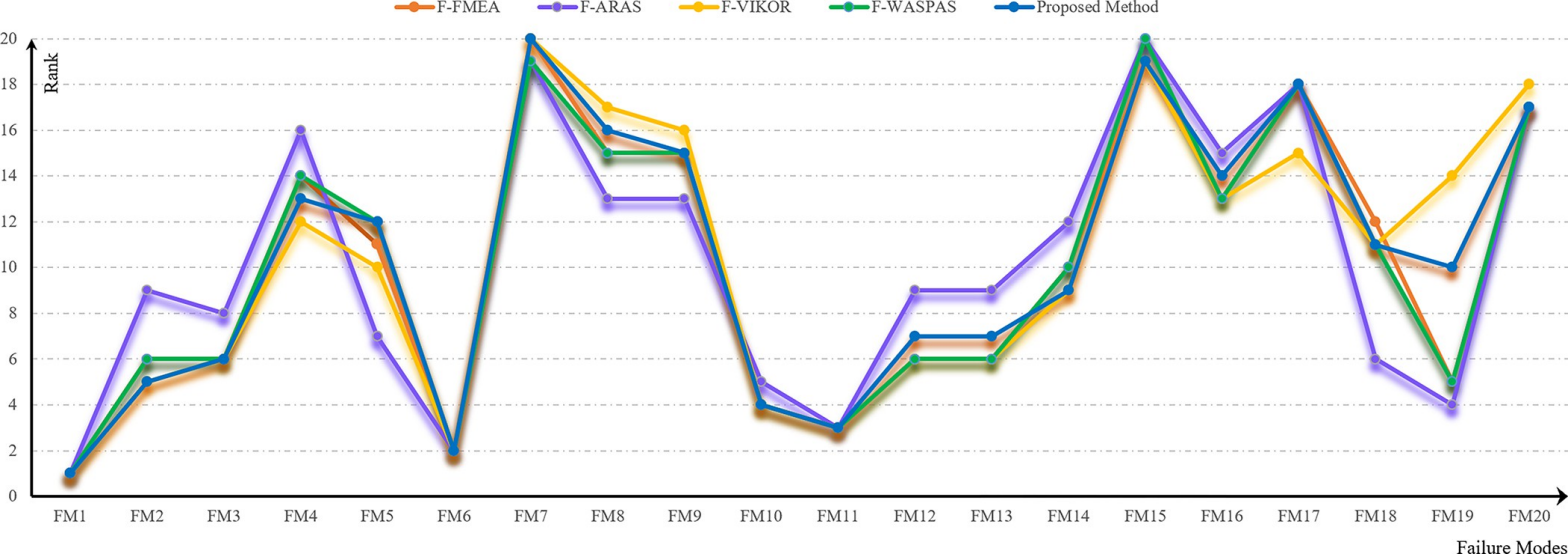
Failure mode ranking obtained using different methods.

Additionally, in Fig 7, rankings derived from the F-FMEA method, three fuzzy decision-making methods, and the proposed approach are presented. Based on Table 9 and Fig 7, the leading 5 risks, garnering the highest scores and exerting a more substantial impact on graft rejection, can be described as follows.

Irregular consumption of anti-rejection medications (FM1) holds the highest ranking in terms of its impact on graft rejection. Anti-rejection medications, also known as immunosuppressants, are employed in the context of organ transplantation or cell transfer. These drugs play a vital role in managing and decreasing immune responses against the transplanted graft, preventing rejection. Progress in anti-rejection drugs has substantially reduced the risk of organ graft rejection. However, it’s significant to note that the likelihood of this occurrence still varies among patients.

Broad-spectrum immunosuppressive medications can lead to a decline in the immune system’s functionality, resulting in a spectrum of adverse effects. Given that each of these medications selectively targets distinct facets of the immune system activation pathways, patients may find themselves necessitating multiple drugs. The immune system is intricately designed to identify foreign tissues upon exposure, triggering diverse immune responses, including the activation of immune cells, and the production and secretion of antibodies. In order to compromise the immune system, which employs a multitude of activation mechanisms for body defense, it becomes imperative to impede these varied activation pathways, compelling patients to adopt a regimen. Novel anti-rejection drugs act by inhibiting one or more immune system activation mechanisms, with this inhibition not exclusively confined to organ transplant rejection. Consequently, there is an augmented risk of infections due to compromised immune activity. Conversely, certain cancers are linked to viruses, contributing to a higher prevalence of cancers, particularly those associated with viruses, in transplant patients undergoing prolonged exposure to these drugs.

Frequent side effects of these medications encompass an elevated susceptibility to high blood pressure, diabetes, and increased blood lipids–all recognized risk factors for cardiovascular diseases. Consequently, cardiovascular diseases are frequently observed or exacerbated in transplant patients. A frequently utilized category of immunosuppressive drugs, entail common side effects such as physical transformations including obesity, diabetes, and osteoporosis.

Given the diminishing risk of rejection beyond the initial year post-transplantation, it becomes imperative for the transplant team to progressively taper the dosage of these medications after the first year to mitigate adverse effects. However, any reduction in drug dosage introduces the inherent risk of organ rejection. The most effective strategy to avert such incidents entails regular check-ups and examinations. Consequently, it is imperative for the transplant team to closely monitor the patient. Regrettably, there is currently no universally accepted scientific method for evaluating the immune system’s activity, leading to a delicate balance between the risk of graft rejection and the potential complications of treatment. Expertise within the transplant team plays a pivotal role in navigating this intricate balance, underscoring the need for physicians on the transplant team to be vigilant in overseeing the patient’s health. Furthermore, a care plan adapted to the unique condition of each patient should be formulated by the relevant medical team.

The second reason (FM6) for on organ rejection is the impact of elapsed ischemia time. The time of ischemia (lack of blood supply) is a crucial factor in the selection and success of organ transplantation. Extended periods of ischemia can cause damage and deterioration of the organ tissues, potentially adversely affecting the ability to accept a new organ. An organ that has undergone a prolonged period of ischemia is likely to have more serious damage to its tissues and cells. Such damage can lead to a decrease in the organ’s functionality and its ability to accept and tolerate a transplant. Increasing the duration of organ ischemia leads to the following:

Extended periods of cold ischemia are associated with a phenomenon known as ischemia-reperfusion injury during organ transplantation. This occurrence involves additional damage to the organ upon the restoration of blood flow, triggering inflammatory responses, oxidative stress, and cellular damage that can impact the overall organ function. Prolonged cold ischemia is commonly linked to an elevated risk of delayed graft function (DGF). DGF manifests as an extended period for the transplanted organ to regain normal function. The extension of cold ischemia durations increases the likelihood of the immune system recognizing and rejecting the transplanted organ. Sensitization of the immune system to antigens during the preservation period raises the risk of an immune response against the graft.

The longer an organ undergoes deprivation of blood flow and oxygen during cold ischemia, the greater the potential for damage to cellular structures. This damage poses implications for the overall viability and function of the transplanted organ, potentially compromising its long-term success.

In the realm of transplant medicine, continuous efforts are directed towards minimizing cold ischemia durations through efficient organ procurement and transportation methods. This involves meticulous control of organ transfer timing and the development of advanced devices simulating the internal body environment to extend organ preservation.

The third reason (FM11) for on organ rejection is skin cancer. People who have previously been exposed to harmful sunlight or other radiation sources, may face an elevated risk of complications associated with organ transplant rejection. Skin cancer is not typically directly associated with organ transplant rejection; however, the therapeutic effects and side effects of skin cancer can impact the organ transplant process. Various treatments for skin cancer, such as surgery, chemotherapy agents, and radiation therapies, may influence the immune system and the body’s ability to accept and sustain the transplanted organ.

In all methods except the F-ARAS method, FM10 is selected as the fourth rank. Problems in the oral cavity, including infections or oral diseases, can have implications for organ transplant rejection. These issues may directly contribute to medical complications following organ transplantation. Dental Infections may lead to heightened immune system activity, and this increased immune response could potentially play a role in confronting transplant organ. Additionally, potential damages and the presence of harmful microorganisms may impact overall health. Here are several oral problems that could potentially impact organ transplant rejection: oral infections, periodontitis (gum inflammation), sunburn in the mouth, oral cancers and etc.

Based on the proposed method, FM12 is taken in the fifth rank. Cytomegalovirus (CMV) is a member of the herpesvirus family and this virus can cause infection in individuals with weakened immune systems and, in some cases, even in those with healthy immune systems. In the context of organ transplantation, CMV can have significant effects. In transplant recipients, the risk of CMV infection increases. This heightened risk is due to the fact that after organ transplantation, the immune system is weakened to prevent organ rejection, providing CMV with a greater opportunity to become active and cause infection. CMV infection in organ transplant recipients can lead to serious complications, including inflammation, kidney problems, and, notably, it can contribute to the rejection of the transplanted organ. The utilization of antiviral medications specifically formulated to counteract CMV is guided by the healthcare provider’s recommendations and continually monitored. For individuals with compromised immune systems, the critical importance of providing supportive care and ensuring proper nutrition cannot be overstressed.

In specific scenarios, additional interventions involving drug administration or methods to improve blood circulation may be essential as supplementary approaches to address severe complications related to CMV.

Tailored treatment plans are necessary for each individual, and decisions regarding therapy should be determined by the treating physician, taking into account the unique characteristics of each patient. Emphasizing the importance of thorough follow-up by the healthcare provider and the treatment team is paramount.

**Fig 8:**
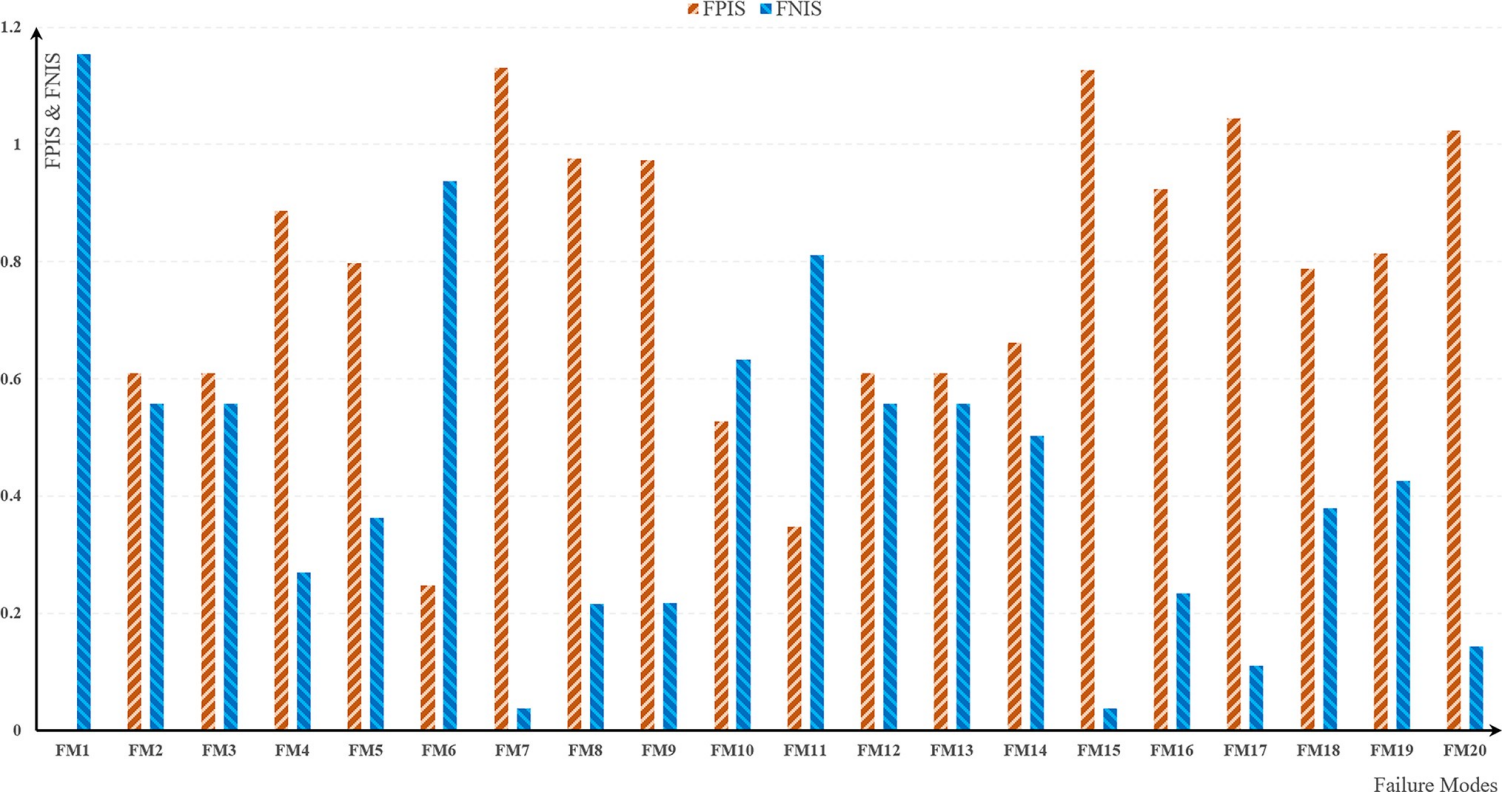
Correlation between failure modes and their respective relative risk levels.

A proposed F-MCDM approach based on F-FMEA, has to offer trustworthy information. It aids decision-makers and designers in understanding failure scenarios in relation to the severity of risks. Given the awareness of high-risk elements and the interventions implemented to ensure post-transplant success, it is necessary to enhance the identification of other risks as depicted in Fig 7, Furthermore, for risks ranked lower, a comprehensive project definition should be established. Additionally, Fig 8, indicates the distance of each fault mode from FPIS, FNIS with computation of Eqs (33) and (34), which represent FPIS and FNIS. Clearly, higher $\psi_{C}^{-}$ value indicates a more perilous risk, warranting increased priority. Conversely, a higher r$\psi_{C}^{+}$ value suggests a lower priority, necessitating classification among low-risk factors.

Differing from the previous notion of employing a singular method for prioritizing failure modes, this paper presents a more comprehensive approach to rank determining factors. The proposed method, considering multiple criteria during risk ranking, should be considered more reliable than a singular approach. As various methods utilize different logics for final ranking, we incorporated the concept of integration to achieve the desired ultimate desirability score. Furthermore, we proceed to examine the reasons for organ rejection and the reduction of graft survival to understand the contributing factors to transplant rejection.

**Fig 9:**
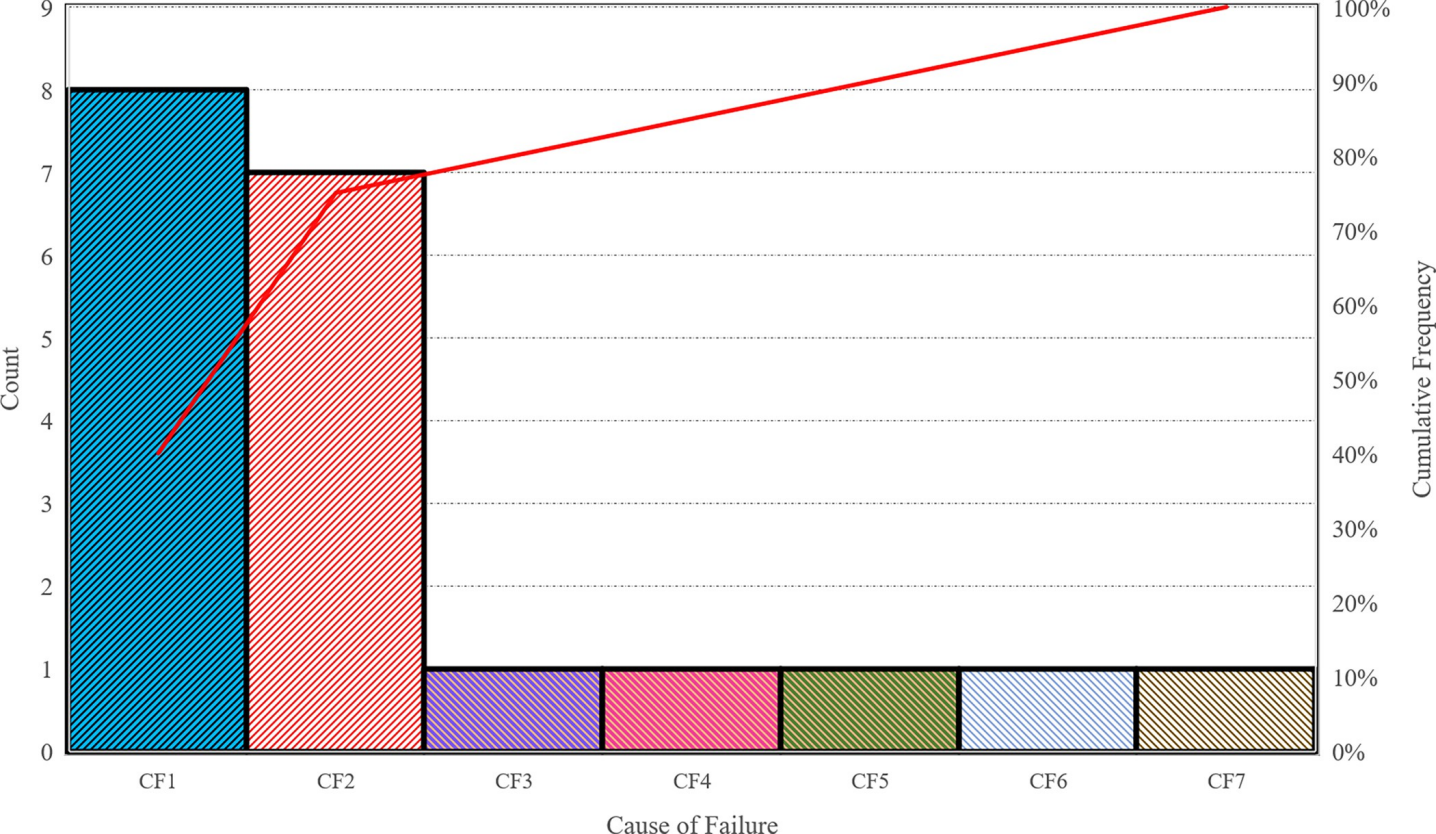
Reference of potentials and danger identified in post-transplant risk assessment.

In Table 10 and Fig 9, 20 hazards are identified, with 8 resulting from failure and negligence on the part of the recipient to follow medical advice and 7 stemming from the use of anti-transplant drugs and compatibility issues with the individual’s body. Furthermore, the subsequent 5 hazards are outlined, encompassing various reasons for their occurrence. The risk assessment is then conducted using Pareto Fig 9 and the Pareto management principle, commonly referred to as the 80:20 principle. According to Pareto’s theory, approximately 80% of events are caused by 20% of factors. The concept of Pareto optimality, which underlines stability and balance, was originally formulated by the Italian economist Pareto [78]. The 80:20 theory was subsequently applied to various human activities, indicating that, for instance, 80% of a product’s sales may come from 20% of customers, and 80% of the profits could originate from 20% of the work. This principle is occasionally referred to as 70–30. Additionally, this rule holds true in the context of post-transplant risk assessment.

**Table 10:** Reference of potentials and danger identified in post-transplant risk assessmen.

Row	Potential Cause	Abundance
1	Failure and negligence on the part of the recipient to follow medical advice	8
2	Taking anti-transplant rejection drugs (immunosuppressants)	7
3	Patients’ conscience agony	1
4	Mismanagement planning and type of organ allocation in network	1
5	Lack of technology to simulate the body environment outside the body	1
6	Weakening of cellular tissues	1
7	Decreased blood flow, dehydration, diet	1

As noted, the rankings generated by different methods show disparities. The key consideration here is that relying solely on the ultimate ranking could result in overlooking vital details, including the varying severity of distinct failure modes in impacting patient survival. Furthermore, it is crucial to subject the results to review by specialized experts in the transplantation field to ensure alignment with real-world post-transplant scenarios. The subsequent section will thoroughly assess the proposed approach.

### 5.4. Model validation

During the validation phase of the proposed model, insights were gathered from three experts, each distinguished in their respective fields: cardiology, nephrology, and hepatology. The opinions of these specialists were quantified and presented in Table 11. To ensure a comprehensive evaluation of risks, additional input was verbally collected from a diverse group of professionals involved in transplant procedures, including an anesthesiologist, an operating room technician, a pulmonary specialist, nurses, and a specialized psychologist. Without exception, all contributors unanimously stressed the importance of the identified 20 criteria for post-transplant patient survival, underscoring the significance of adhering to each of these factors. The model’s results and its reliability underwent a comprehensive verbal evaluation grounded in 20 case scenarios. It’s noteworthy that, recognizing the potential variance in experts’ opinions, ranging from full concurrence to disagreement, which could be expressed verbally as slightly agreeable or in a 50–50 stance, we applied fuzzy theory and the Likert scale. The Likert scale, commonly utilized in social science research and surveys, facilitated the measurement of attitudes, opinions, and perceptions of the specialists toward the model’s outcomes [79]. The Likert scale allows researchers to quantify subjective opinions and attitudes in a standardized and measurable way [80]. Subsequently, we recorded the perspectives of each expert in the Table 11, categorized as optimistic, neutral, and pessimistic. Since triangular fuzzy numbers are represented as (*α*,*β*,*γ*), we have that in order fuzziness, employed Eq (36) for its defuzzification [81].


$$x_{m}=\frac{\alpha +4\beta +\gamma }{6}$$ (36)

**Table 11:** Opinions of experts regarding each of the reasons for failure mode.

Expert Number	Cause of failure	Optimistic	Neutral	Pessimistic	Defuzzification
Expert 1	FM1	5	7	9	7
	FM2	3	5	5	4.67
	FM3	3	5	5	4.67
	FM4	1	3	3	2.67
	FM5	1	3	5	3
	FM6	5	7	7	6.67
	FM7	1	3	3	2.67
	FM8	1	3	3	2.67
	FM9	1	3	3	2.67
	FM10	3	5	9	5.33
	FM11	3	7	9	6.67
	FM12	3	5	7	5
	FM13	3	5	5	4.67
	FM14	3	3	5	3.33
	FM15	1	1	3	1.33
	FM16	1	3	3	2.67
	FM17	1	1	3	1.33
	FM18	1	3	5	3
	FM19	3	3	5	3.33
	FM20	1	1	3	1.33

Expert Number	Cause of failure	Optimistic	Neutral	Pessimistic	Defuzzification
Expert 2	FM1	7	7	9	7.33
	FM2	3	5	5	4.67
	FM3	3	3	4	3.17
	FM4	1	1	3	1.33
	FM5	1	3	5	3
	FM6	5	7	7	6.67
	FM7	1	1	1	1
	FM8	1	1	3	1.33
	FM9	1	1	3	1.33
	FM10	3	5	9	5.33
	FM11	5	5	9	5.67
	FM12	3	5	7	5
	FM13	3	5	5	4.67
	FM14	1	3	5	3
	FM15	1	1	1	1
	FM16	1	3	3	2.67
	FM17	1	1	1	1
	FM18	1	3	5	3
	FM19	3	5	7	5
	FM20	1	1	1	1
Expert 3	FM1	5	9	9	8.33
	FM2	5	5	9	5.67
	FM3	3	5	9	5.33
	FM4	3	5	5	4.67
	FM5	3	5	5	4.67
	FM6	7	7	9	7.33
	FM7	1	1	3	1.33
	FM8	1	3	5	3
	FM9	1	3	3	2.67
	FM10	5	7	7	6.67
	FM11	5	7	9	7
	FM12	5	7	7	6.67
	FM13	5	5	9	5.67
	FM14	3	5	9	5.33
	FM15	1	1	1	1
	FM16	3	3	4	3.17
	FM17	1	3	3	2.67
	FM18	3	5	7	5
	FM19	3	5	7	5
	FM20	1	3	3	2.67

Table 11, expert’s opinions have been converted from qualitative to quantitative using the Likert scale (see Appendix 2 & Table 13 in S1 File) in optimistic, neutral, and pessimistic conditions, and we have the following:

This paper outlines the viewpoints of three experts on 20 factors influencing transplant rejection or diminished graft survival in patients. The opinions of each expert are presented in the Table 10, reflecting their individual perspectives. As evident in the mentioned Table 11, the lack of discipline in taking anti-transplant drugs (FM1) is highlighted as the primary cause of graft damage and the leading factor in transplant rejection. Unanimously, all three experts concur on this issue. Furthermore, factors such as prolonged ischemic time (FM6) are identified as critical determinants of success or failure in graft transplantation, according to the insights of our expert panel. In other instances, analyze their significance based on the figures provided in the last column. For instance, FM3 involves violating quarantine rules after transplantation.

According to expert opinions, there is often no necessity for strict quarantine in such cases, though it could be a contributing factor. FM4, concerning post-transplant infection due to errors in pre-transplant medical research, has become less common with the advancements in laboratory equipment precision. The controversy among experts regarding the donor’s keratin level suggests that, for some, it may be the primary reason for transplant rejection and even organ loss. Lastly, FM7, which pertains to engaging in strenuous activity post-transplant, is not regarded as a significant factor in transplant rejection. Nevertheless, it may exert its influence, and broader examinations should be conducted on it.

**Fig 10:**
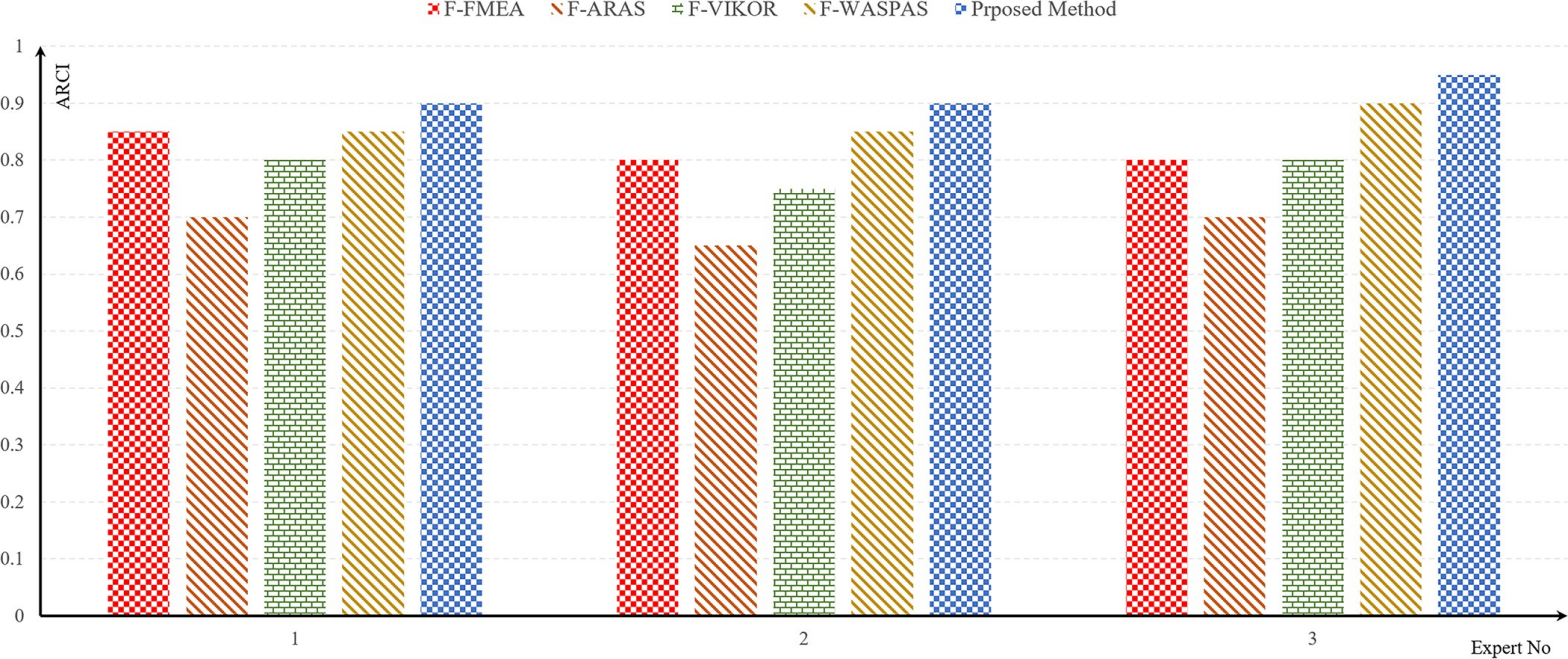
The nearness of expert perspectives in five methods.

Additionally, considering Table 11, we compared the proximity of the results of the methods presented in this paper in the previous sections with the opinions of each expert in Fig 10.

The Approach Results Convergence Index (ARCI) in the column Fig 10 indicates the level of proximity between the output of each method and expert opinions. The higher ARCI value, employed method holds more credibility. Consequently, implying the method’s results are more reliable.

In Fig 10, we observe the experts’ rankings of impactful risks on patient survival. The comparison involves assessing the alignment of expert opinions with the outputs of the employed methods for each risk. The proposed method exhibits the highest degree of conformity with the experts’ opinions. On average, there is a 91.67% similarity between expert opinions and the outcomes of the proposed method. Following that, the F-WASPAS method, on average, secures the second position in the rankings, demonstrating a mean proximity to expert perspective of 86.67%. Ranked third, fourth, and fifth are the F-FMEA, F-VIKOR, and F-ARAS methods, respectively. Consequently, the obtained results from the evaluations indicate the superiority of the proposed method.

## 6. Discussions

The objective of this research is to identify the risks and hazards linked to organ transplant surgeries. The F-FMEA, F-ARAS, F-VIKOR. F-WASPAS method with was the proposed hybrid based on F-FMEA method employed to assess the existing risks. The use of a fuzzy approach in this paper is adopted to consider linguistic uncertainty and linguistic variables. A total of 20 significant risks were identified through a combination of literature review and expert opinions. Subsequently, a questionnaire was developed to evaluate three input parameters: the likelihood of occurrence, severity of effect, and the likelihood of numerical detection, utilizing fuzzy logic. The outcomes of the F-FMEA were presented in Table 6, F-ARAS, F-VIKOR. F-WASPAS method were presented in Table 7 and finally outcomes of the proposed method in Table 8.

The risks have been prioritized according to the intensity of the threat and their more significant impact on the survival of patients for each cause and effect, experts in the field recommended an appropriate method for preventing and correcting the root causes of failure.

The findings indicated that the most critical risk was the absence of a lack of discipline in taking anti-transplant drugs and it secures the first rank among the identified risks. lack of discipline in taking anti-transplant drugs can have a significant impact on the survival of the transplanted organ and the overall health of the transplant recipient. Strict adherence to the prescribed medication regimen is vital for preventing rejection and maintaining the long-term success of organ transplantation. Patients should work closely with their healthcare team to address any concerns, side effects, or challenges related to medication adherence. In the second rank, the ischemic time of the transplanted organ has elapsed is recognized as a highly influential factor in diminishing the quality and lower effectiveness of the transplanted organ. Considering that time is among the most crucial parameters in the organ allocation network, redesign, the design of equitable systems, and efficient allocation methods can significantly mitigate this issue in the organ transplant network. Adhering to personal hygiene, not smoking, maintaining a healthy diet, and timely vaccination are essential practices. Transplant recipients, like everyone else, need regular physical activity as a sedentary lifestyle can pose risks for them. Any form of physical activity that provides energy can be beneficial. Walking, cycling, swimming, tennis, and yoga are typically suggested for transplant patients. Consulting with the transplant team can help in choosing the most suitable exercise. Exercise plays a crucial role in improving their physical well-being and reducing stress levels. Transplant recipients need to engage in regular physical activity to enhance overall health and quality of life, under the guidance of their healthcare team. Among all, the utmost importance lies in individuals who have undergone organ transplantation should maintain open communication with their healthcare team. The approach to managing the risk of cancer post-transplant is highly personalized, and strategies may be adjusted based on factors such as the patient’s overall health, the type of organ transplanted, and specific risk factors. Consistent attendance at follow-up appointments and adherence to recommended screenings are essential aspects of post-transplant care. Furthermore, for other risks that contribute to a decrease in patient survival most common risk was cardiovascular disease, caused by the side effects of drugs (immunosuppressants). Table 6 highlights elevated risks associated with diabetes, cancer, viral infections, Cytomegalovirus, oral diseases, and physical dysfunction among organ transplant recipients due to the incompatibility with anti-transplant drugs. Strict adherence to quarantine rules is imperative for patients, given their weakened and sensitive immune systems for 3–5 months post-transplant, where even a minor illness can pose a significant threat. Factors such as medical errors, nursing oversights, blood donor creatine levels, and neglect of the ischemia time of the transplanted organ can compromise its quality, leading to rejection and potential fatality. Rigorous testing by diverse teams is crucial to minimize errors and ensure both the donor and recipient are in optimal physical condition. Strenuous activities and pregnancy should be avoided for 6–9 months after organ transplantation due to physical weakness and reduced capacity. Mental health challenges, including impatience, fear, and depression, are common among patients and should be addressed through medical counseling sessions and positive activities.

Failure to adhere to the prescribed diet by the doctor can complicate the treatment process and elevate the risk of organ rejection. Additionally, as of now, technology has not devised a method to accurately replicate the in vivo environment within the body, impacting the quality of in vitro transplants. Post-transplant physical dysfunction is a commonly observed occurrence among transplant patients. This condition is primarily attributed to the side effects of anti-transplant drugs and a decline in self-confidence resulting from changes in self-image, leading to diminished interest in sexual activity. Consulting with a physician is advisable for the use of specific medications, such as those for blood pressure, seizures, and antidepressants, to determine the appropriate dosage and avoid medication rejection. Establishing a supportive relationship with one’s spouse and seeking guidance from a counselor or treating physician can help mitigate the adverse effects of this condition.

Age plays a crucial role in the success of transplantation, as statistics reveal a higher risk of failure for individuals aged 50 compared to those aged 65. Additionally, various studies underscore the importance of age compatibility between donors and recipients in the literature, proposing that a 20 to 40-year age difference is optimal for both parties. Nevertheless, experts hold diverse opinions on this matter, although they unanimously agree that preoperative testing of middle-aged patients can mitigate complications.

According to Table 10 and Fig 9, 75% of transplantation-related issues stem from two primary causes, manageable through correct intervention for a safe, non-surgical procedure. Eight of the 20 identified risks are attributed to failure and negligence on the part of the recipient to follow medical advice, while seven are linked to the side effects of anti-transplant drugs (immunosuppressants) and taking anti-transplant rejection drugs. Regular follow-ups, typically every two weeks, prove essential in gauging medication effectiveness. These follow-ups enable experts to adjust drug types or dosages promptly, effectively managing complications in the process.

## 7. Conclusions and future research directions

In recent decades, there has been notable progress in surgical procedures and pharmacological interventions. Despite these advancements, the frequency of organ transplants has experienced a decline, while the demand for organs persists at a level significantly surpassing the available supply. Organ transplantation stands out as a pivotal therapeutic approach for various diseases within the medical landscape. Therefore, it becomes imperative to meticulously identify the factors contributing to diminished survival rates and the rejection of transplanted organs. This thorough identification is crucial for implementing more robust and targeted control measures, a matter of paramount importance in the field of organ transplantation. Investigation of the recent literature has highlighted a gap in acknowledging a comprehensive array of factors that lead to transplant rejection and diminished post-transplant survival.

The FMEA method is a systematic process designed for identifying potential errors. However, in real-world applications, numerical values might not precisely represent the assessments made by decision-makers or FMEA team members, who frequently use linguistic variables to evaluate risk indicators like probability, severity, and detection. To mitigate these limitations, the fuzzy method is proposed as a flexible approach. This study not only aims to resolve this issue through the F-FMEA method but also endeavors to overcome the constraints of F-FMEA methods, striving for more comprehensive and reliable results in the analysis. In this paper, we introduce a fuzzy hybrid MCDM approach based on F-FMEA to identify and rank the risks affecting the survival and quality-adjusted life years of the patient.

In the ranking obtained, factors like non-adherence to anti-transplant drugs (immunosuppressants), the elapse of ischemic time of the transplanted organ, and the risk of cancer, particularly skin cancer and lymphoma, hold positions 1 to 3. Additionally, the paper output indicated that 8 out of the 20 identified risks are attributed to the recipient’s non-compliance with medical recommendations due to failure and negligence. Meanwhile, 7 cases while seven are linked to the side effects of anti-transplant drugs (immunosuppressants) and taking anti-transplant rejection drugs. Additionally, in this paper was observed that diverse decision-making methods, employing varied logics and approaches, yielded identical outcomes when applied to identical data inputs. The suggested approach seeks to enhance robustness and reliability by amalgamating the underlying logics of disparate methods. This methodology enables swift assessments by decision-making units and experts of prevailing systems, facilitating the prompt formulation of improvement strategies. In summary, the notable innovations and achievements of this paper encompass:

(a) utilizing the FMEA methodology and MCDM method for the ranking of post-transplant risks to improve both survival and quality life years from transplant, (b) developing a novel hybrid MCDM approach based on F-FMEA for the risk ranking process, (c) providing a fuzzy logic approach to tackle inherent uncertainties in input parameters, (d) identification and ranking of risks, along with the presentation of preventive solutions and identification and ranking of risks, along with the presentation of preventive solutions.

In conclusion, to evaluate the proposed method, we ranked the identified high-risk scenarios using the expertise and experience of three knowledgeable individuals. Subsequently, we compared this ranking with F-FMEA, F-ARAS, F-VIKOR and F-WASPAS methods. The observation revealed that the proposed approach exhibits the highest level of alignment compared to other methods.

Several potential directions for future research are as follows:

I. Expanding on this research can involve conducting separate investigations for each of the mentioned causes within hospitals, comparing them with hospitals that have successfully addressed these issues in their operational domain. This comparative analysis could involve statistical evaluations of the outcomes.
II. Further exploration in this domain could delve deeper into data mining techniques to uncover additional factors influencing success, such as blood group relevance in transplantation, and to achieve more precise age estimations for successful operations.
III. Future research could explore alternative MCDM methods, for example, COPRAS and ELECTRE, alongside the previously mentioned methods. The integration of various methodologies could be considered for comparative analysis in upcoming studies.
IV. For forthcoming studies, it is advisable to conduct a thorough and rational weighting of indicators for risk assessment with FMEA, utilizing methodologies like BWM and DEMATEL.

## Supporting information

S1 File (DOCX)
