# Supplementary material for: Risk assessment of organ transplant operation: A fuzzy hybrid MCDM approach based on fuzzy FMEA
Source: PLoS One. 2024 May 23;19(5):e0299655. doi: 10.1371/journal.pone.0299655 (PMC11115332; doi:10.1371/journal.pone.0299655)
Supplement: S1 File — (DOCX) [file pone.0299655.s001.docx]

Appendix 1

Table 12. Linguistic variables for RPN in the F-FMEA approach

| Detectable | | | | | Effect Severity | | | | | | | | | | Probability of Occurrence | | | | | | Potential failure | Row |
| --- | --- | --- | --- | --- | --- | --- | --- | --- | --- | --- | --- | --- | --- | --- | --- | --- | --- | --- | --- | --- | --- | --- |
| **Remote** | **Low** | **Moderate** | **High** | **Very High** | **Very Minor** | **Minor** | **Very Low** | **Low** | **Moderate** | **High** | **Very High** | **Dangerous with warning** | **Dangerous without warning** | **Very High** | | **High** | **Moderate** | **Low** | **Remote** |  | |  |
|  |  |  |  |  |  |  |  |  |  |  |  |  |  |  | |  |  |  |  | The ischemic time of the transplanted organ has elapsed | | 1 |
|  |  |  |  |  |  |  |  |  |  |  |  |  |  |  | |  |  |  |  | Improper diet | | 2 |
|  |  |  |  |  |  |  |  |  |  |  |  |  |  |  | |  |  |  |  | Mental illness | | 3 |
|  |  |  |  |  |  |  |  |  |  |  |  |  |  |  | |  |  |  |  | Error in medical research | | 4 |
|  |  |  |  |  |  |  |  |  |  |  |  |  |  |  | |  |  |  |  | Donor blood creatinine levels | | 5 |
|  |  |  |  |  |  |  |  |  |  |  |  |  |  |  | |  |  |  |  | Lack of discipline in taking anti-transplant drugs | | 6 |
|  |  |  |  |  |  |  |  |  |  |  |  |  |  |  | |  |  |  |  | Do heavy work after transplantation | | 7 |
|  |  |  |  |  |  |  |  |  |  |  |  |  |  |  | |  |  |  |  | Ignoring quarantine rules | | 8 |
|  |  |  |  |  |  |  |  |  |  |  |  |  |  |  | |  |  |  |  | Possible cardiovascular disease | | 9 |
|  |  |  |  |  |  |  |  |  |  |  |  |  |  |  | |  |  |  |  | Probability of developing diabetes | | 10 |
|  |  |  |  |  |  |  |  |  |  |  |  |  |  |  | |  |  |  |  | Risk of cancer | | 11 |
|  |  |  |  |  |  |  |  |  |  |  |  |  |  |  | |  |  |  |  | Possibility of infection and virus | | 12 |
|  |  |  |  |  |  |  |  |  |  |  |  |  |  |  | |  |  |  |  | The possibility of contracting cytomegalovirus | | 13 |
|  |  |  |  |  |  |  |  |  |  |  |  |  |  |  | |  |  |  |  | The possibility of developing oral diseases | | 14 |
|  |  |  |  |  |  |  |  |  |  |  |  |  |  |  | |  |  |  |  | Becoming pregnant shortly following the transplant | | 15 |
|  |  |  |  |  |  |  |  |  |  |  |  |  |  |  | |  |  |  |  | Recipient and donor age | | 16 |
|  |  |  |  |  |  |  |  |  |  |  |  |  |  |  | |  |  |  |  | Decreased quality of transplanted organ outside the body | | 17 |
|  |  |  |  |  |  |  |  |  |  |  |  |  |  |  | |  |  |  |  | Impaired physical function of the body after transplantation | | 18 |
|  |  |  |  |  |  |  |  |  |  |  |  |  |  |  | |  |  |  |  | Nursing mistakes | | 19 |
|  |  |  |  |  |  |  |  |  |  |  |  |  |  |  | |  |  |  |  | Inadequate physical activity and limited mobility | | 20 |

Appendix 2

| **Cost Perspective** | **Quantitative Scale** | **Profit Perspective** |
| --- | --- | --- |
| Very High | 1 | Very Low |
| High | 3 | Low |
| Moderate | 5 | Moderate |
| Low | 7 | High |
| Very Low | 9 | Very High |

Table 13. Likert scale
